# Comparative effectiveness and safety of immunotherapeutic strategies in ovarian cancer: a systematic review and network meta-analysis

**DOI:** 10.3389/fonc.2025.1659897

**Published:** 2025-11-10

**Authors:** Xinyao Wang, Yuli Zhang, Wei Xie, Haiying Liu, Lianghui Cui, Min Feng

**Affiliations:** Department of Gynecology, Dongzhimen Hospital of Beijing University of Chinese Medicine, Beijing, China

**Keywords:** ovarian cancer, immunotherapy, cancer vaccines, immune checkpoint inhibitors, network meta-analysis

## Abstract

**Background:**

Ovarian cancer remains the most lethal gynecologic malignancy, with poor survival despite standard therapies. Immunotherapy represents a promising option, yet the comparative efficacy and safety among different immunotherapies are unclear. This network meta-analysis aimed to evaluate and rank multiple immunotherapeutic strategies for ovarian cancer.

**Methods:**

A systematic search of PubMed, Embase, Medline, PsycINFO, Cochrane Central Register of Controlled Trials, and Web of Science was performed through May 31, 2025. Randomized controlled trials (RCTs) comparing immunotherapies were included. Outcomes were overall survival (OS), progression-free survival (PFS), objective response rate (ORR), disease control rate (DCR), treatment-related adverse events (TRAEs), and grade ≥3 adverse events. Bayesian network meta-analysis was conducted using random-effects models, calculating standardized mean differences (SMDs) or mean differences (MDs) with 95% confidence intervals (CIs) for continuous variables, and odds ratios (ORs) with 95% CIs for categorical variables.

**Results:**

Twenty-six RCTs involving 5,982 patients were included. Cancer vaccines (CV) (HR = 0.56, 95% CI 0.43–0.73) and dual immune checkpoint blockade (DICB) (HR = 0.65, 95% CI 0.46–0.92) significantly improved OS compared with controls. CV also prolonged PFS (SMD = 0.95, 95% CI 0.16–1.75). CTLA-4 inhibitors markedly increased ORR (OR = 99.32, 95% CI 1.18–8360.43), though no significant DCR differences were observed. PD-1 inhibitors demonstrated the best safety profile, reducing grade ≥ 3 AEs (OR = 0.16, 95% CI 0.08–0.33) and overall TRAEs versus other immunotherapies.

**Conclusion:**

CV and DICB yielded the most consistent survival benefits, while PD-1 inhibitors showed superior safety. These findings support tailored, biomarker-informed immunotherapy approaches and combination strategies to optimize efficacy and tolerability in ovarian cancer. Further head-to-head trials are warranted to confirm these results.

**Systematic Review Registration:**

PROSPERO (CRD420251083861).

## 1 Introduction

Ovarian cancer is a malignant neoplasm originating predominantly from the surface epithelium of the ovary and remains the most lethal gynecologic cancer worldwide. Unlike other solid tumors, ovarian cancer typically presents with nonspecific symptoms and is diagnosed at an advanced stage in over 70% of cases, contributing to poor long-term prognosis (1, 2). Globally, there were an estimated 314,000 new ovarian cancer cases and 207,000 related deaths in 2020, ranking it among the top ten causes of female cancer mortality (3). The burden is expected to rise significantly, with projections indicating over 446,000 new cases and 313,000 deaths annually by 2040 (4). Despite advances in diagnosis and treatment, the overall 5-year survival rate remains below 50%, and for advanced-stage disease, it drops below 30%. These figures reflect not only the biological aggressiveness of the disease but also substantial socioeconomic consequences, including high healthcare costs and productivity losses. The global economic burden of ovarian cancer has been estimated at over $70 billion annually, driven largely by early mortality and limited therapeutic success (5).

The standard treatment paradigm for ovarian cancer includes cytoreductive surgery followed by platinum-based chemotherapy, with or without targeted maintenance therapy such as bevacizumab or poly (ADP-ribose) polymerase (PARP) inhibitors (6, 7). While these treatments can achieve high initial response rates, the majority of patients relapse, often within two years of completing frontline therapy, and become resistant to further chemotherapy (8). This cycle of recurrence underscores the need for novel treatment modalities capable of delivering durable responses. Immunotherapy, a modality designed to restore or enhance the immune system’s ability to eliminate cancer cells, has transformed the treatment landscape in several malignancies, including melanoma, non-small cell lung cancer, and renal cell carcinoma. In ovarian cancer, immunotherapeutic approaches under investigation include immune checkpoint blockade (targeting programmed cell death protein 1 (PD-1), programmed death-ligand 1 (PD-L1), or cytotoxic T-lymphocyte–associated protein 4 (CTLA-4)), therapeutic cancer vaccines (CVs), adoptive cell therapies such as tumor-infiltrating lymphocytes (TILs) or chimeric antigen receptor T cells (CAR-T cells), and oncolytic viruses (9, 10). These modalities offer mechanistically distinct strategies that aim to overcome the immunosuppressive tumor microenvironment characteristic of ovarian cancer.

Early-phase clinical trials evaluating immune checkpoint inhibitors in ovarian cancer have shown modest efficacy, with reported objective response rates (ORR) ranging from 4% to 15% (11). These limited results are often attributed to a low tumor mutational burden and an immunologically “cold” tumor microenvironment, which impairs T-cell infiltration and activation (12). In response, combination strategies—such as checkpoint blockade with chemotherapy, anti-angiogenic agents, or PARP inhibitors—are being explored to enhance therapeutic efficacy (13). Several systematic reviews and meta-analyses have assessed the outcomes of PD-1/PD-L1 inhibitors or specific immune combinations in ovarian cancer, reporting modest improvements in PFS or ORR but increased toxicity profiles (14, 15). However, the comparative efficacy and safety of the full range of available immunotherapeutic strategies remain unclear. Most existing meta-analyses focus on single interventions and lack head-to-head comparisons between different immunotherapy modalities, leaving clinicians uncertain about the optimal treatment strategy for different patient populations.

In this context, network meta-analysis (NMA) provides an efficient and rigorous methodological framework to synthesize both direct and indirect evidence across multiple interventions. By enabling the comparative evaluation of diverse therapies—even in the absence of direct comparisons—NMA is particularly suited for evaluating rapidly evolving fields such as cancer immunotherapy (16). Therefore, this study aimed to conduct a comprehensive systematic review and Bayesian network meta-analysis to assess and compare different immunotherapeutic strategies in ovarian cancer. This research addresses a critical gap in evidence, with the potential to inform clinical decision-making, support future trial design, and improve outcomes for women affected by this aggressive malignancy.

## 2 Methods

This systematic review and network meta-analysis was conducted following the Preferred Reporting Items for Systematic Reviews and Meta-Analyses (PRISMA) statement and the PRISMA extension for network meta-analysis guidelines (PRISMA-NMA) (17, 18). Given the nature of this systematic review and meta-analysis, ethical approval or informed consent was not required.

### 2.1 Data sources and searches

A comprehensive literature search was performed across PubMed, Medline, Embase, PsycINFO, the Cochrane Central Register of Controlled Trials, and Web of Science from their inception until May 31, 2025. The search strategy utilized Medical Subject Headings (MeSH) and text words related to ovarian cancer (“ovarian cancer,” “peritoneal cancer,” “fallopian tube cancer”), immunotherapy (“immunotherapy,” “checkpoint inhibitor,” “programmed cell death protein 1 (PD-1),” “programmed death-ligand 1 (PD-L1),” “cytotoxic T-lymphocyte–associated protein 4 (CTLA-4)”), and randomized controlled trials (RCTs), combined using Boolean operators “AND” and “OR”.

For transparency, the complete PubMed search string is presented below: ((“ovarian cancer”[MeSH Terms] OR “ovarian neoplasms”[Title/Abstract] OR “peritoneal cancer”[Title/Abstract] OR “fallopian tube cancer”[Title/Abstract]) AND (“immunotherapy”[MeSH Terms] OR “immunotherapy”[Title/Abstract] OR “checkpoint inhibitor”[Title/Abstract] OR “PD-1”[Title/Abstract] OR “PD-L1”[Title/Abstract] OR “CTLA-4”[Title/Abstract])) AND (“randomized controlled trial”[Publication Type] OR “randomized”[Title/Abstract] OR “randomised”[Title/Abstract] OR “RCT”[Title/Abstract])). Database-specific search strategies for Medline, Embase, PsycINFO, Cochrane Central, and Web of Science remain available in **Supplementary File 1** for full reproducibility.

Additionally, references from all included studies and systematic reviews published within the past five years were reviewed to identify further relevant articles. Two independent reviewers screened titles, abstracts, and full texts, with disagreements resolved by discussion or consultation with a third reviewer.

### 2.2 Study selection

Studies meeting the following inclusion criteria were considered eligible: (1) Population: patients aged ≥18 years with histologically confirmed ovarian cancer, including primary peritoneal and fallopian tube cancers; (2) Intervention: immunotherapy-based treatments administered in the experimental group; (3) Comparator: usual care, placebo, standard chemotherapy, radiotherapy, or different immunotherapeutic strategies in head-to-head network comparisons; (4) Outcomes: clearly reported clinical efficacy and safety outcomes; (5) Study design: randomized controlled trials; and (6) published in English.

Studies were excluded based on the following criteria: (1) patients with ovarian cancer combined with other cancer types; (2) intervention and control groups both using identical non-immunotherapy strategies or identical immunotherapy regimens; (3) unclear description of treatment protocols; (4) absence of relevant outcome data or failure to obtain data from authors after repeated requests; (5) non-randomized studies, conference abstracts, study protocols, reviews, meta-analyses, and case reports. Two independent reviewers assessed eligibility by reviewing titles, abstracts, and full texts.

### 2.3 Data extraction

Eligible studies were managed using EndNote X9 software to avoid duplication. Two independent reviewers extracted study characteristics, including publication details (authors, publication year), patient demographics (age, cancer type), treatment protocols (intervention regimens, comparator regimens, and treatment duration), and outcome measures. Missing means and standard deviations were estimated based on guidelines from the Cochrane Handbook (19). When means and SDs were imputed, calculations were derived from reported medians, ranges, or interquartile ranges using validated statistical formulas. Each imputed value was subsequently cross-checked for plausibility against the original descriptive statistics provided in trial reports to minimize potential error. If required data were unavailable from published sources, corresponding authors were contacted at least four times over six weeks. If no response was received, studies were excluded. Data from studies with multiple experimental arms using identical interventions were pooled for analyses.

### 2.4 Risk of bias assessment

Risk of bias for each included RCT was independently assessed by two reviewers using the revised Cochrane Risk of Bias Tool (RoB 2), evaluating five domains: randomization process, deviations from intended interventions, missing outcome data, measurement of outcomes, and selection of reported results (20). Disagreements were resolved through consultation with a third reviewer.

### 2.5 Data coding

Immunotherapeutic interventions from included studies were categorized and coded into the following groups: Cancer vaccines (CV), CTLA-4 inhibitors, Dual immune checkpoint blockade (DICB), IDO1 inhibitors, Immunostimulants (IS), PD-1 inhibitors, PD-L1 inhibitors, PD-L1 inhibitors combined with cancer vaccines (PD-L1+CV), and Radioimmunotherapy (RIT). Standard chemotherapy, radiotherapy, targeted therapy, or placebo were collectively grouped as control (CON).

### 2.6 Outcome measures

Primary outcomes evaluated were overall survival (OS), progression-free survival (PFS), objective response rate (ORR), disease control rate (DCR), treatment-related adverse events (TRAEs), and grade ≥3 adverse events (≥3 AEs). OS and PFS were analyzed exclusively as time-to-event outcomes and expressed as hazard ratios (HRs) with 95% confidence intervals (CIs). Reported HRs and corresponding standard errors were directly extracted from trial publications; when HRs were not available, they were reconstructed from published survival curves using validated methods (21). ORR and DCR represented the proportion of patients experiencing complete or partial response and stable disease or better, defined primarily according to the Response Evaluation Criteria in Solid Tumors (RECIST v1.1); when studies explicitly reported immune-related criteria, immune-related RECIST (irRECIST) was applied (22). TRAEs included any adverse event attributed to treatment, while ≥3 AEs encompassed serious or severe adverse events requiring clinical intervention, graded according to the Common Terminology Criteria for Adverse Events (CTCAE, version 4.0 or higher, most commonly v4.0 or v5.0).

### 2.7 Data analysis

Statistical analyses were conducted using R software (version XX, R Foundation for Statistical Computing, Vienna, Austria) with the “netmeta” package in addition to Stata software version 17.0 (StataCorp LLC, College Station, TX, USA). Network meta-analysis (NMA) was employed to compare the efficacy and safety of different immunotherapies for ovarian cancer. Network diagrams were constructed to visualize direct and indirect comparisons among interventions. Considering anticipated clinical heterogeneity, a random-effects model was utilized to incorporate within- and between-study variability.

For OS and PFS, hazard ratios (HRs) with 95% CIs were synthesized using the netmeta package, which provides a frequentist framework suitable for time-to-event data. For categorical outcomes (e.g., ORR, DCR, TRAEs, ≥3 AEs), odds ratios (ORs) with 95% CIs were used. Statistical heterogeneity was assessed using the I² statistic, categorized as low (≤25%), moderate (50%), and high (≥75%). All network meta-analyses were conducted under a frequentist framework, and prior references to Bayesian methods have been removed for clarity. The Surface Under the Cumulative Ranking Curve (SUCRA) was calculated to rank treatments, with higher SUCRA values indicating better relative performance. Publication bias was assessed visually through adjusted funnel plots and statistically via Egger’s test, with a p-value <0.05 indicating potential bias (23). Predictive interval plots were generated to explore heterogeneity further and assess the variability in treatment effects. All statistical tests were two-sided, with statistical significance set at a p-value <0.05. No significant global inconsistency was detected; detailed results of inconsistency models are provided in **Supplementary File 5**.

## 3 Results

### 3.1 Characteristics of included studies

**Figure 1:**
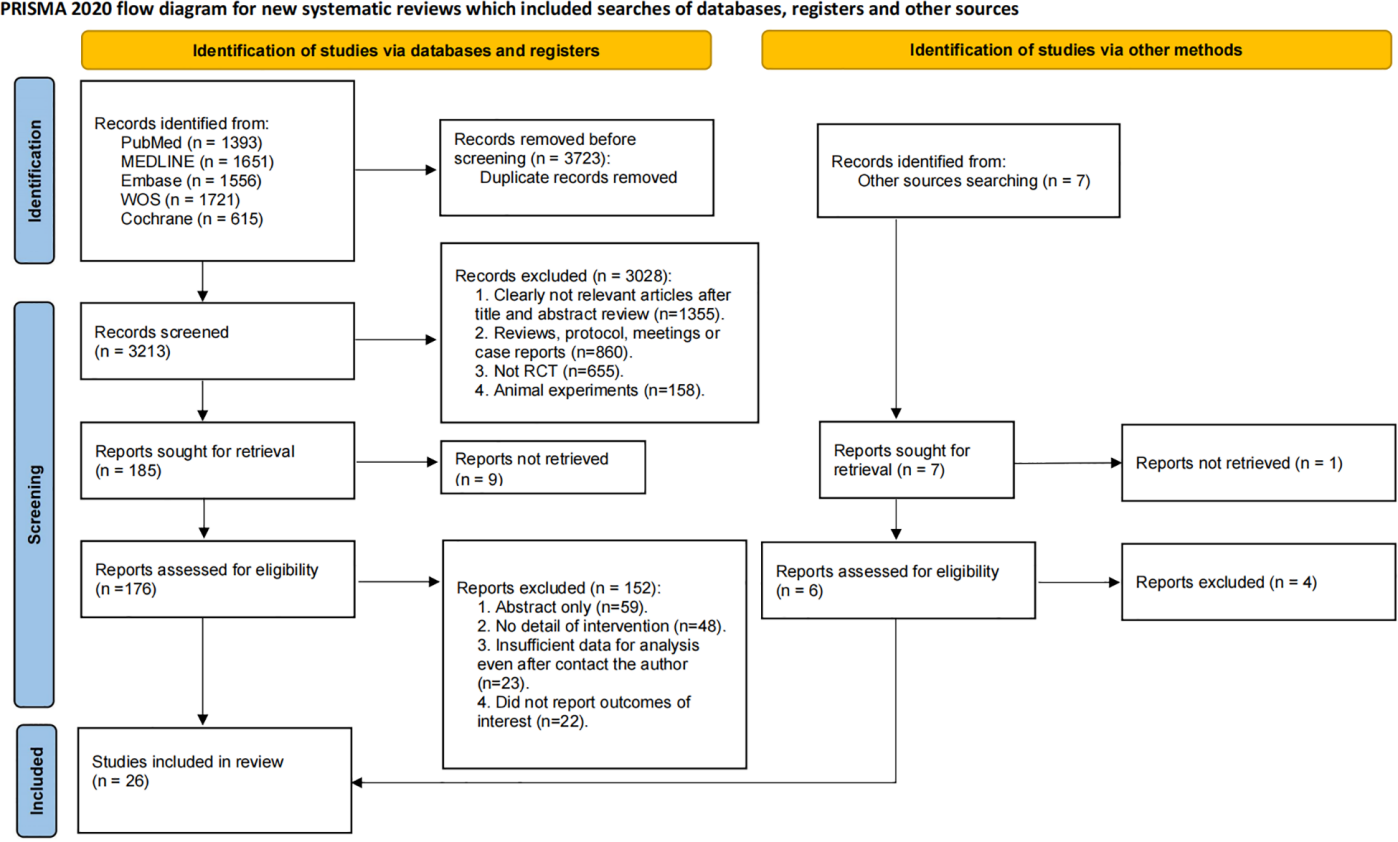
PRISMA Flow diagram of the search process for studies.

A total of 6,936 records were initially identified through systematic database searches. After removing 3,723 duplicates, 3,213 records underwent title and abstract screening. Subsequently, 3,028 records were excluded, and the remaining 185 articles underwent full-text review. Ultimately, 26 RCTs involving 5,982 ovarian cancer patients met eligibility criteria and were included in the systematic review and network meta-analysis (**Figure 1**) (24–41).

The included studies were published between 1980 and 2024, with a median publication year of 2021. Among these, 20 trials were open-label RCTs, and 7 were double-blind. Sample sizes ranged from 21 to 1,301 participants, with a median sample size of 97. The mean age of participants ranged from 54.0 to 63.9 years, with a median of 60.0 years.

Regarding immunotherapeutic interventions, eight studies utilized CV, two employed DICB, one evaluated IDO1 inhibitors, three used IS, three evaluated PD-1 inhibitors, eight involved PD-L1 inhibitors, two combined PD-L1 + CV, one used RIT, and one employed CTLA-4 inhibitors. Twenty-three studies included conventional therapies such as chemotherapy, radiotherapy, or targeted therapy as CON. Detailed characteristics of the included studies are presented in **Supplementary File 2**.

### 3.2 Results of network meta-analysis

#### 3.2.1 Overall survival

A total of 18 RCTs with 4,645 ovarian cancer patients were included for OS. The network plot of direct and indirect comparisons is presented in **Figure 2.1**. According to SUCRA rankings (**Figure 3.1**), the top three regimens associated with the greatest reduction in mortality risk were CV (94.6%), DICB (82.8%), and PD-L1 inhibitors (58.7%), whereas CON (22.8%) ranked lowest. As shown in **Table 1.1**, CV (HR = 0.56, 95% CI: 0.43–0.73) and DICB (HR = 0.65, 95% CI: 0.46–0.92) significantly reduced the risk of death compared with CON. Moreover, CV showed superiority over IS (HR = 0.56, 95% CI: 0.38–0.82), PD-1 inhibitors (HR = 0.66, 95% CI: 0.47–0.93), and PD-L1 inhibitors (HR = 0.69, 95% CI: 0.49–0.98).

**Figure 2:**
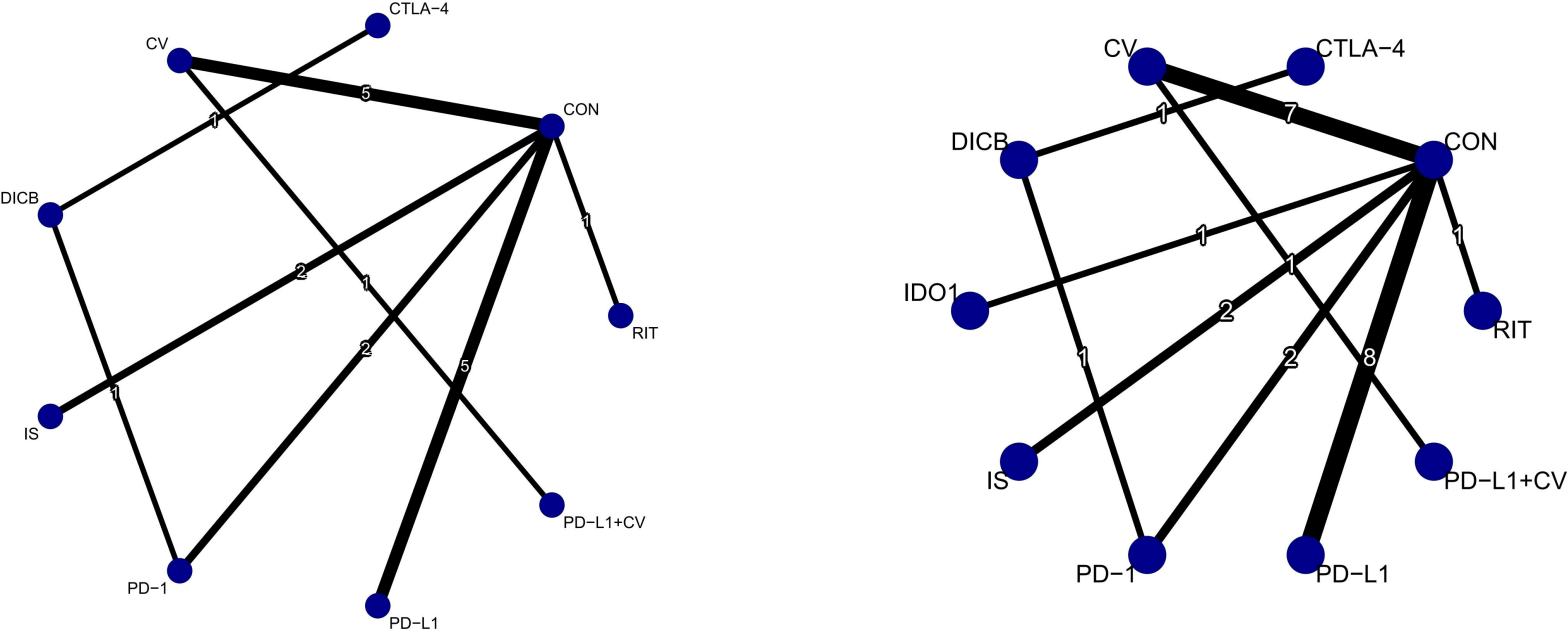
Network plots of efficacy outcomes (1. OS; 2. PFS). Node size reflects sample size; edge thickness indicates number of direct comparisons. CV, cancer vaccines; DICB, dual immune checkpoint blockade; CTLA-4, cytotoxic T-lymphocyte–associated protein 4 inhibitors; PD-1, programmed cell death protein 1 inhibitors; PD-L1, programmed death-ligand 1 inhibitors; IS, immunostimulants; IDO1, indoleamine 2,3-dioxygenase 1 inhibitors; RIT, radioimmunotherapy; CON, control.

**Figure 3:**
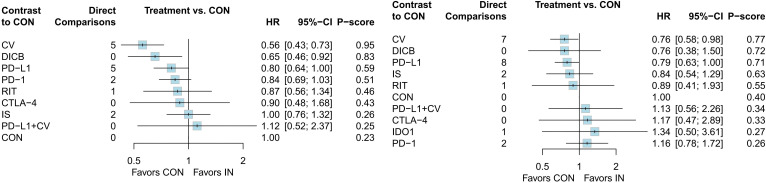
Forest plots of efficacy outcomes (1. OS; 2. PFS).

Table 1League table of efficacy and safety outcomes in ovarian cancer patients.

**Table:** 

Table 1.1 OS													
CV													
0.85 (0.55; 1.32)	DICB												
0.69 (0.49; 0.98)	0.81 (0.54; 1.22)		PD-L1										
0.66 (0.47; 0.93)	0.78 (0.59; 1.02)		0.95 (0.71; 1.29)		PD-1								
0.64 (0.38; 1.07)	0.75 (0.43; 1.30)		0.92 (0.57; 1.50)		0.97 (0.60; 1.56)	RIT							
0.62 (0.31; 1.24)	0.73 (0.43; 1.24)		0.90 (0.46; 1.75)		0.94 (0.52; 1.71)	0.97 (0.45; 2.09)	CTLA-4						
0.56 (0.38; 0.82)	0.65 (0.42; 1.01)		0.80 (0.56; 1.15)		0.84 (0.60; 1.19)	0.87 (0.52; 1.45)	0.90 (0.45; 1.78)		IS				
0.50 (0.25; 1.01)	0.59 (0.26; 1.34)		0.72 (0.33; 1.58)		0.76 (0.35; 1.65)	0.78 (0.33; 1.86)	0.80 (0.30; 2.15)		0.90 (0.40; 2.00)	PD-L1+CV			
0.56 (0.43; 0.73)	**0.65 (0.46; 0.92)**		0.80 (0.64; 1.00)		0.84 (0.69; 1.03)	0.87 (0.56; 1.34)	0.90 (0.48; 1.68)		1.00 (0.76; 1.32)	1.12 (0.52; 2.37)		CON	

Table 1.2 PFS													
CV													
1.00 (0.48; 2.07)	DICB												
0.95 (0.67; 1.34)	0.95 (0.47; 1.95)		PD-L1										
0.90 (0.54; 1.51)	0.91 (0.40; 2.04)		0.95 (0.58; 1.56)		IS								
0.85 (0.38; 1.92)	0.85 (0.30; 2.38)		0.89 (0.40; 2.00)		0.94 (0.39; 2.28)	RIT							
0.76 (0.58; 0.98)	0.76 (0.38; 1.50)		0.79 (0.63; 1.00)		0.84 (0.54; 1.29)	0.89 (0.41; 1.93)	CON						
0.67 (0.35; 1.27)	0.67 (0.25; 1.78)		0.71 (0.34; 1.46)		0.74 (0.33; 1.68)	0.79 (0.28; 2.23)	0.89 (0.44; 1.78)		PD-L1+CV				
0.65 (0.25; 1.67)	0.65 (0.36; 1.19)		0.68 (0.27; 1.74)		0.72 (0.26; 1.97)	0.76 (0.23; 2.52)	0.86 (0.35; 2.13)		0.97 (0.31; 3.04)	CTLA-4			
0.56 (0.20; 1.57)	0.57 (0.17; 1.88)		0.59 (0.21; 1.64)		0.62 (0.21; 1.84)	0.66 (0.19; 2.33)	0.75 (0.28; 2.01)		0.84 (0.25; 2.82)	0.87 (0.23; 3.34)	IDO1		
0.65 (0.41; 1.05)	0.65 (0.38; 1.14)		0.69 (0.44; 1.08)		0.72 (0.40; 1.30)	0.77 (0.32; 1.83)	0.86 (0.58; 1.28)		0.97 (0.44; 2.16)	1.01 (0.44; 2.28)	1.16 (0.40; 3.36)		PD-1

Table 1.3 ORR													
CTLA-4													
6.64 (0.13,348.85)	CV												
8.95 (0.36,224.71)	1.35 (0.13,13.51)		DICB										
16.34 (0.42,632.15)	2.46 (0.48,12.59)		1.83 (0.33,10.25)		PD-L1								
26.96 (0.63,1159.36)	4.06 (0.63,26.00)		3.01 (0.43,20.94)		1.65 (0.57,4.74)	IS							
27.88 (0.74,1054.51)	4.20 (0.86,20.40)		3.11 (0.58,16.66)		1.71 (1.14,2.56)	1.03 (0.39,2.74)	CON						
29.33 (0.87,985.86)	4.42 (0.71,27.50)		3.28 (0.81,13.32)		1.80 (0.66,4.90)	1.09 (0.29,4.15)	1.05 (0.42,2.64)		PD-1				
36.14 (0.65,1994.60)	5.44 (0.53,55.45)		4.04 (0.37,43.94)		2.21 (0.39,12.69)	1.34 (0.19,9.51)	1.30 (0.24,7.09)		1.23 (0.18,8.51)	PD-L1 + CV			
99.32 (1.18,8360.43)	14.96 (0.75,298.05)		11.09 (0.53,232.78)		6.08 (0.46,79.61)	3.68 (0.24,55.97)	3.56 (0.28,45.18)		3.39 (0.23,50.45)	2.75 (0.13,58.39)			IDO1

Table 1.4 DCR													
DICB													
1.76 (0.35,8.87)	PD-L1												
2.13 (0.45,10.10)	1.21 (0.79,1.86)		CON										
3.08 (0.57,16.53)	1.75 (0.17,17.99)		1.44 (0.15,14.24)		CTLA-4								
3.84 (0.36,41.01)	2.18 (0.35,13.69)		1.80 (0.30,10.74)		1.25 (0.07,22.78)	PD-L1 + CV							
3.48 (0.99,12.20)	1.98 (0.71,5.47)		1.63 (0.65,4.09)		1.13 (0.14,9.21)	0.91 (0.12,6.75)	PD-1						
4.32 (0.38,48.53)	2.45 (0.37,16.43)		2.02 (0.32,12.92)		1.40 (0.07,26.71)	1.12 (0.09,14.75)	1.24 (0.16,9.82)		IDO1				

Table 1.5 TRAEs													
PD-1													
0.51 (0.00,191.92)	CTLA-4												
0.31 (0.01,14.94)	0.61 (0.01,54.34)	DICB											
0.24 (0.04,1.29)	0.47 (0.00,219.83)	0.76 (0.01,51.61)	CON										
0.24 (0.00,28.32)	0.47 (0.00,935.99)	0.76 (0.00,354.02)	1.00 (0.01,86.96)	IS									
0.23 (0.02,2.27)	0.45 (0.00,257.98)	0.74 (0.01,65.71)	0.97 (0.21,4.49)	0.97 (0.01,109.24)	CV								
0.21 (0.01,4.52)	0.41 (0.00,323.02)	0.67 (0.00,93.11)	0.88 (0.07,11.39)	0.88 (0.01,151.53)	0.91 (0.05,17.83)	IDO1							
0.16 (0.02,1.10)	0.31 (0.00,157.43)	0.51 (0.01,38.17)	0.67 (0.26,1.71)	0.67 (0.01,63.94)	0.68 (0.11,4.12)	0.76 (0.05,11.53)		PD-L1					
0.05 (0.00,1.66)	0.10 (0.00,96.71)	0.17 (0.00,29.71)	0.22 (0.01,4.47)	0.22 (0.00,47.93)	0.22 (0.01,4.26)	0.25 (0.00,12.95)		0.33 (0.01,7.74)	PD-L1 + CV				

Table 1.6 ≥3 AEs													
PD-1													
0.50 (0.18,1.45)	DICB												
0.36 (0.06,1.98)	0.71 (0.18,2.72)	CTLA-4											
0.21 (0.09,0.51)	0.42 (0.11,1.65)	0.59 (0.09,4.03)	CV										
0.16 (0.08,0.33)	0.32 (0.09,1.16)	0.45 (0.07,2.91)	0.77 (0.48,1.24)	CON									
0.17 (0.03,0.84)	0.33 (0.05,2.28)	0.47 (0.04,4.90)	0.80 (0.18,3.45)	1.03 (0.25,4.34)	PD-L1 + CV								
0.15 (0.06,0.39)	0.30 (0.07,1.25)	0.42 (0.06,3.00)	0.72 (0.33,1.54)	0.93 (0.51,1.70)	0.90 (0.19,4.28)	IS							
0.12 (0.06,0.27)	**0.24 (0.06,0.90)**	0.34 (0.05,2.24)	0.58 (0.33,1.01)	0.75 (0.56,1.00)	0.72 (0.17,3.13)	0.80 (0.41,1.56)	PD-L1						
0.04 (0.01,0.28)	**0.08 (0.01,0.72)**	0.11 (0.01,1.48)	0.18 (0.03,1.23)	0.24 (0.04,1.51)	0.23 (0.02,2.38)	0.25 (0.04,1.77)	0.32 (0.05,2.06)		IDO1				

Bold values: statistically significant.

#### 3.2.2 Progression-free survival

For PFS, 24 RCTs involving 5,904 patients were analyzed. The corresponding network structure is shown in **Figure 2.2**. SUCRA rankings (**Figure 3.2**) identified CV (77.1%), DICB (72.4%), and PD-L1 inhibitors (71.1%) as the most effective strategies in reducing progression risk, while PD-1 inhibitors (26.4%) ranked lowest. However, as indicated in **Table 1.2**, no significant differences were observed among the treatment comparisons.

#### 3.2.3 Objective response rate

Seventeen studies involving 3,193 ovarian cancer patients were included to evaluate ORR. The network plot for direct comparisons is displayed in **Figure 4.1**. Based on SUCRA values (**Figure 5.1**), the top three immunotherapies enhancing ORR were CTLA-4 (94.1%), CV (79.8%), and DICB (72.1%), with IDO1 ranking lowest (12.6%). **Table 1.3** shows a significant improvement in ORR with CTLA-4 compared to IDO1 (OR = 99.32, 95% CI = 1.18 to 8360.43).

**Figure 4:**
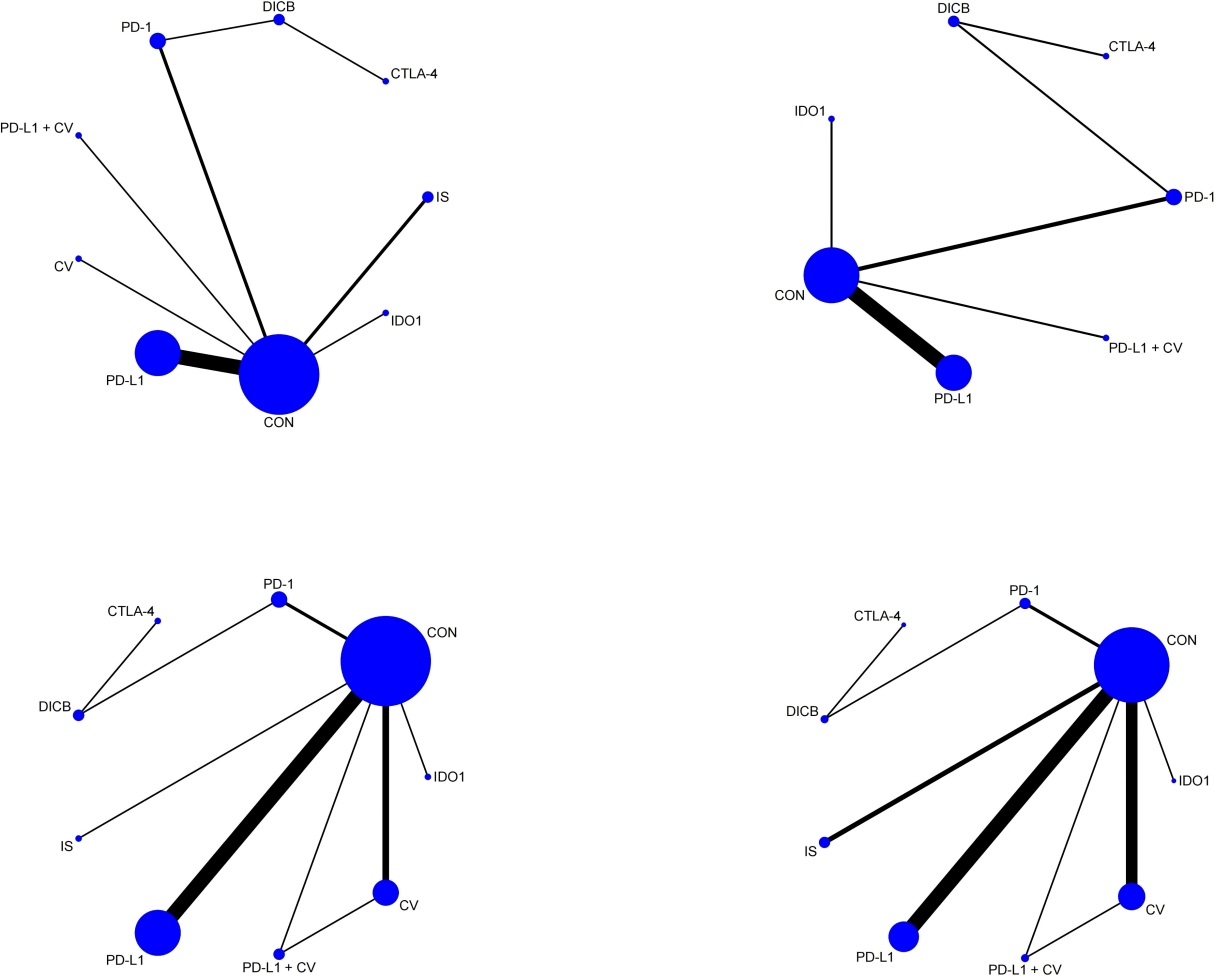
Network plots of secondary outcomes (1. ORR; 2. DCR; 3. TRAEs; 4. ≥3 AEs). Node and edge definitions as in **Figure 2**.

**Figure 5:**
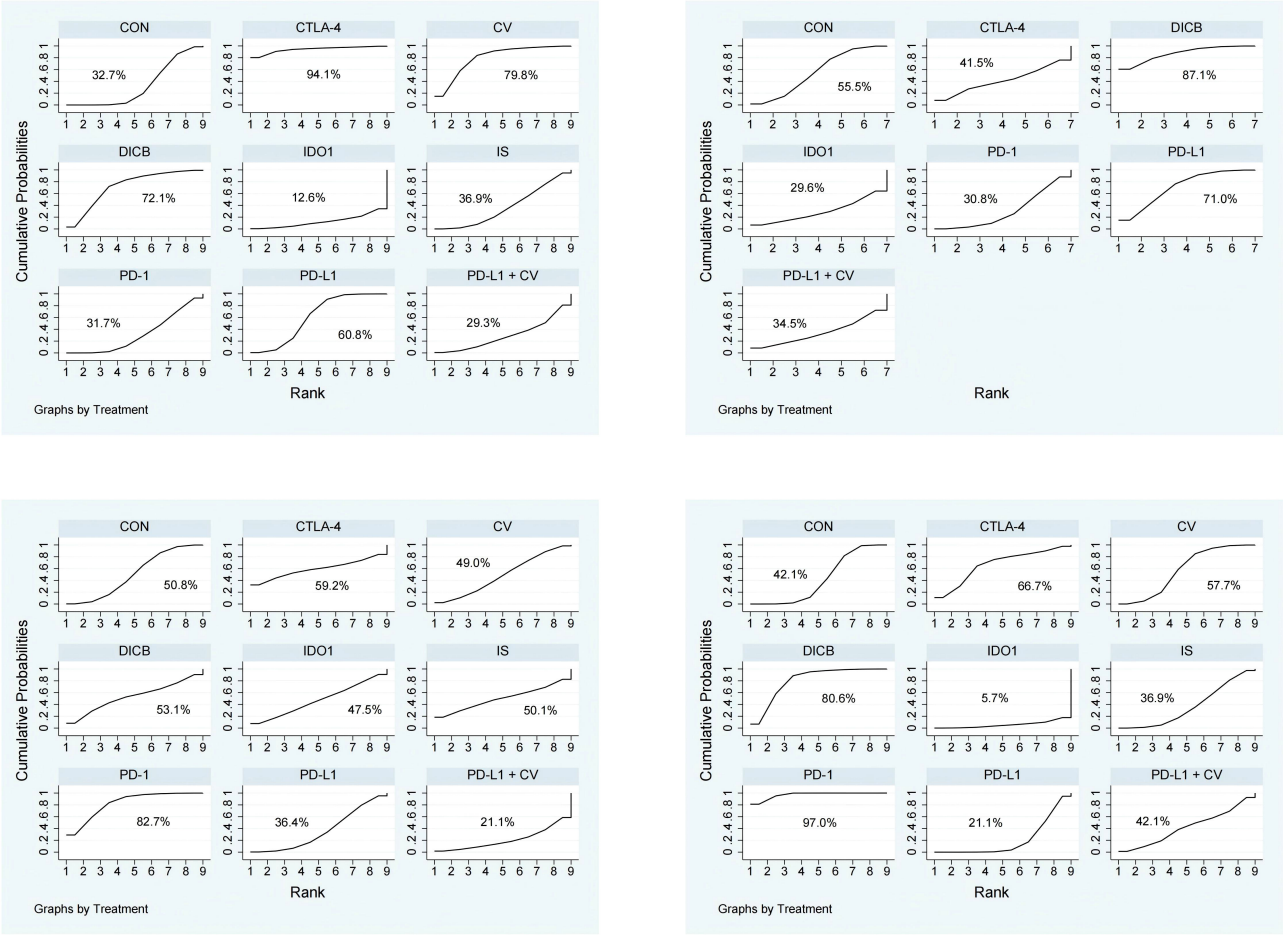
SUCRA probability ranking plots of secondary outcomes (1. ORR; 2. DCR; 3. TRAEs; 4. ≥3 AEs). Higher SUCRA values indicate better relative ranking.

#### 3.2.4 Disease control rate

Twelve studies involving 2,548 ovarian cancer patients assessed DCR. The network plot is presented in **Figure 4.2**. SUCRA rankings (**Figure 5.2**) indicated the highest efficacy for DICB (87.1%), followed by PD-L1 (71.0%) and CTLA-4 (41.5%), with IDO1 ranked lowest (29.6%). However, as indicated in **Table 1.4**, no significant differences were identified between groups (P>0.05).

#### 3.2.5 Treatment-related adverse events

Twenty studies involving 4,793 ovarian cancer patients evaluated TRAEs. **Figure 4.3** displays the network comparisons. According to SUCRA rankings (**Figure 5.3**), PD-1 (82.7%), CTLA-4 (59.2%), and DICB (53.1%) were associated with the lowest incidence of TRAEs, while PD-L1 + CV ranked lowest (21.1%). Nevertheless, no significant differences were observed among the groups (P>0.05, **Table 1.5**).

#### 3.2.6 Grade ≥3 adverse events

Twenty-five studies involving 5,388 ovarian cancer patients assessed grade ≥3 AEs. **Figure 4.4** shows network comparisons. SUCRA rankings (**Figure 5.4**) indicated that PD-1 (97.0%), DICB (80.6%), and CTLA-4 (66.7%) most effectively minimized grade ≥3 AEs, whereas IDO1 ranked lowest (5.7%). As detailed in **Table 1.6**, PD-1 significantly reduced grade ≥3 AEs compared to CV (OR = 0.21, 95% CI = 0.09 to 0.51), CON (OR = 0.16, 95% CI = 0.08 to 0.33), PD-L1 + CV (OR = 0.17, 95% CI = 0.03 to 0.84), IS (OR = 0.15, 95% CI = 0.06 to 0.39), PD-L1 (OR = 0.12, 95% CI = 0.06 to 0.27), and IDO1 (OR = 0.04, 95% CI = 0.01 to 0.28). Furthermore, DICB significantly reduced ≥3 AEs compared to PD-L1 (OR = 0.24, 95% CI = 0.06 to 0.90) and IDO1 (OR = 0.08, 95% CI = 0.01 to 0.72).

### 3.3 Risk of bias and publication bias

Among the 26 included RCTs, 12 were rated as low risk of bias, 11 with some concerns, and 3 as high risk overall. For randomization, 18 trials were low risk, 6 had some concerns, and 2 were high risk. Regarding deviations from intended interventions, 21 trials were low risk, 3 had some concerns, and 2 had high risk. Missing outcome data posed low risk in 17 studies, some concerns in 7, and high risk in 2. For outcome measurement, 25 were low risk and 1 had some concerns. All trials had low risk for selective reporting (**Supplementary File 3**).

Publication bias was evaluated using funnel plots (**Supplementary File 4**). All funnel plots (S4.1–S4.6) showed varying degrees of asymmetry, suggesting potential bias. However, Egger’s tests indicated p > 0.05 for all outcomes, suggesting no significant publication bias among the included studies.

## 4 Discussion

This comprehensive network meta-analysis incorporated 26 RCTs with a total of 5,982 patients with ovarian cancer, systematically comparing the relative efficacy and safety of multiple immunotherapeutic strategies. Three principal findings emerged from this analysis. First, CV and DICB demonstrated the most favorable performance in prolonging OS and PFS, significantly reducing the risk of death and delaying disease progression. Second, CTLA-4 inhibitors and cancer vaccines achieved notable improvements in ORR, whereas enhancements in DCR were limited and showed no statistically significant differences among treatment groups. Third, PD-1 inhibitors and DICB were associated with lower incidences of TRAEs and≥3 AEs, indicating comparatively favorable tolerability. Collectively, these findings provide comprehensive comparative evidence on the hierarchy of immunotherapeutic efficacy and safety in ovarian cancer, offering a valuable reference for personalized treatment selection and for the design of future immunotherapy trials.

OS and PFS are the most essential endpoints in ovarian cancer, as they reflect both the life-prolonging efficacy of treatment and the persistence of disease control. In this network meta-analysis, CV and DICB showed the most consistent advantages across both OS and PFS, while PD-L1 and CTLA-4 inhibitors also appeared to provide potential benefits. These findings are in partial agreement with previous studies suggesting that immune checkpoint combination strategies and vaccine-based immunotherapy may enhance clinical outcomes in advanced or recurrent ovarian cancer. For example, dual inhibition of PD-1 and CTLA-4 has yielded durable survival benefits in several solid tumors, and similar patterns have begun to emerge in ovarian cancer trials (42). Likewise, vaccine-based approaches such as oregovomab have demonstrated the capacity to trigger tumor-specific immune activation, leading to delayed progression and prolonged survival (28). The superior performance of CV and DICB may be explained by their complementary mechanisms of immune activation. DICB amplifies antitumor responses by releasing T cells from multiple inhibitory pathways, thereby strengthening both immune priming and effector function (43). CV promotes antigen-specific cytotoxic T-cell responses and maintains immune surveillance capable of suppressing residual disease and preventing relapse (44). CTLA-4 inhibition further enhances these effects by reducing regulatory T-cell–mediated suppression and supporting the expansion of activated effector cells within the tumor microenvironment (45). These coordinated immunologic effects may underlie the dual advantage of CV and DICB in prolonging survival and delaying disease progression. The findings highlight the therapeutic promise of integrating checkpoint blockade with tumor vaccines as a means of achieving sustained immune control in ovarian cancer.

Beyond survival-related endpoints, ORR and DCR remain important surrogate measures in ovarian cancer, as they provide insight into the ability of therapies to induce measurable tumor regression or stabilize disease burden. These metrics are clinically relevant in assessing whether a treatment can meaningfully shrink tumor lesions or halt disease progression, especially for patients who have limited options after recurrence. In this network meta-analysis, CTLA-4 inhibitors and cancer vaccines demonstrated a clear advantage in improving ORR, suggesting these modalities may elicit a stronger cytotoxic T-cell–mediated antitumor response capable of achieving radiographically detectable tumor shrinkage. However, their impact on DCR appeared more modest, with no statistically significant differences across treatment groups. This divergence may be explained by the immunologically “cold” phenotype of many ovarian cancers, characterized by a suppressive tumor microenvironment with high densities of regulatory T cells, myeloid-derived suppressor cells, and limited tumor-infiltrating lymphocytes. Such an environment may restrict the durability of response after initial tumor regression, limiting improvements in DCR even when ORR is enhanced (46). Moreover, immunotherapies may require a longer period to establish a sustained disease control effect than is typically captured in conventional trial endpoints, further complicating DCR comparisons (47). These findings suggest that while some immunotherapeutic strategies can robustly induce tumor shrinkage, their capacity to maintain stable disease over time warrants further investigation, and future studies should incorporate immunologically relevant biomarkers to better predict durable disease control.

Safety remains a cornerstone consideration in the management of ovarian cancer, given patients’ frequent exposure to multiple lines of cytotoxic chemotherapy and the cumulative toxicities associated with standard treatments. In this network meta-analysis, PD-1 inhibitors and DICB demonstrated the most favorable safety profiles, with lower risks of treatment-related adverse events and grade ≥3 adverse events compared to other immunotherapeutic modalities. This observation is partly consistent with previous clinical experience showing that PD-1–targeted agents are generally well tolerated in solid tumors, with a lower incidence of severe immune-related toxicities than CTLA-4 blockade alone or combined with other immunostimulatory strategies (48). The superior tolerability of PD-1 inhibitors may reflect their mechanism of action, which selectively restores exhausted effector T-cell activity without provoking broad systemic immune activation, thereby reducing off-target autoimmune responses (49). For DICB, although our pooled analysis suggested a relatively favorable safety profile, this stands in contrast to the broader literature. A plausible explanation is that some included DICB regimens applied modified dosing strategies or selective patient enrollment, potentially mitigating toxicity. Moreover, heterogeneity in AE grading and reporting may have further influenced comparative results. Accordingly, these safety findings for DICB should be regarded as exploratory and require confirmation in future head-to-head trials with harmonized safety endpoints. These mechanistic insights underscore the evolving landscape of immunotherapy, where carefully designed regimens can maximize antitumor efficacy while minimizing immune-related adverse events. Taken together, while PD-1 inhibitors can be considered consistently safe across solid tumors, the safety profile of DICB in ovarian cancer remains uncertain and should be interpreted with caution.

The findings of this network meta-analysis have important clinical implications for ovarian cancer management. By systematically comparing multiple immunotherapeutic strategies, this study provides a comparative framework highlighting treatment options based on relative efficacy and safety. While SUCRA values illustrate the likelihood of favorable performance, they should be viewed as supportive rather than definitive evidence, particularly when effect estimates were not statistically significant (e.g., categorical OS, DCR). In such cases, high rankings may reflect network structure or indirect comparisons rather than true clinical benefit. CV and DICB showed the most favorable survival outcomes across both overall and progression-free survival, whereas PD-1 inhibitors demonstrated the lowest risk of treatment-related and severe adverse events. These findings emphasize the need to balance efficacy with safety and support individualized immunotherapy selection to optimize clinical benefit. This aligns with current therapeutic trends favoring the integration of immune checkpoint blockade, cancer vaccination, and combination regimens to overcome resistance and recurrence. Incorporating molecular and immune profiling may further enable precise patient stratification and guide rational immunotherapy combinations for ovarian cancer.

This study has several strengths. First, to our knowledge, it represents one of the most comprehensive network meta-analyses to date comparing multiple immunotherapeutic modalities in ovarian cancer, integrating both direct and indirect evidence across a large sample size. Second, it applied a rigorous Bayesian framework and prespecified methodological criteria to ensure robust and transparent synthesis of the evidence. However, certain limitations should be acknowledged. First, the included trials displayed considerable heterogeneity in patient populations, disease stages, and previous treatment histories, which may confound pooled estimates. Second, although most included RCTs were judged at low or moderate risk of bias, a minority were rated high risk in domains such as randomization procedures and missing outcome data. These high-risk trials may have contributed uncertainty to pooled effect estimates and relative treatment rankings, particularly in evidence networks with limited direct comparisons. Thus, the interpretation of some results should be made with caution. Third, the relatively small number of head-to-head randomized comparisons among immunotherapies limited the strength of conclusions about relative rankings. Fourth, although several funnel plots exhibited some visual asymmetry, Egger’s tests did not indicate statistical significance (all p > 0.05). This apparent discrepancy may arise because Egger’s regression has limited statistical power when only a small number of studies contribute to each comparison. In addition, asymmetry in funnel plots can be driven by heterogeneity or small-study effects rather than true publication bias. Accordingly, these results should be interpreted with caution: the lack of statistical significance does not definitively exclude the presence of selective reporting, while observed asymmetry does not necessarily confirm bias. Fifth, several studies were excluded because the required outcome data could not be retrieved despite repeated attempts to contact study authors. This may have introduced bias if unpublished or incompletely reported results systematically differed from those included, and thus the possibility of selective availability of evidence should be considered when interpreting our findings. Finally, restricting our search to English-language publications may have introduced language bias, potentially excluding relevant studies reported in other languages. In addition, although missing data were imputed according to Cochrane Handbook recommendations (e.g., estimating standard deviations from other summary statistics), such imputation carries inherent uncertainty and could have influenced pooled estimates and treatment rankings. These two factors—language restriction and data imputation—should therefore be regarded as important limitations when interpreting the robustness and generalizability of our findings. Future prospective trials directly comparing diverse immunotherapy regimens, ideally incorporating predictive biomarkers and standardized outcome definitions, are warranted to validate and extend these results.

## 5 Conclusion

This network meta-analysis of 26 randomized trials involving 5,982 ovarian cancer patients showed that cancer vaccines and dual immune checkpoint blockade achieved the best outcomes in overall and progression-free survival, while CTLA-4 inhibitors and cancer vaccines improved response rates, and PD-1 inhibitors demonstrated the greatest safety. These findings provide comparative evidence to inform immunotherapy selection and support the development of combination and biomarker-guided strategies to optimize ovarian cancer treatment.

## Data Availability

The original contributions presented in the study are included in the article/**Supplementary Material**. Further inquiries can be directed to the corresponding author.
