# Supplementary material for: Comparative effectiveness and safety of immunotherapeutic strategies in ovarian cancer: a systematic review and network meta-analysis
Source: Front Oncol. 2025 Nov 10;15:1659897. doi: 10.3389/fonc.2025.1659897 (PMC12640866; doi:10.3389/fonc.2025.1659897)
Supplement: Supplementary file 1 [file DataSheet1.docx]

**Supplementary**

Table of Contents:

[Supplementary 1: Search Strategy 2](#_Toc159076731)

[Supplementary 2: Characteristics of studies and subjects included in the review 4](#_Toc159076743)

[Supplementary 3: Risk of Bias](#_Toc159076747) 16

[Supplementary 4: Funnel Plots of Publication Bias](#_Toc159076747) 18

[Supplementary 5: Forest plot 2](#_Toc159076747)2

# Supplementary 1: Search Strategy

***Search Strategy:***

| #8 | Search: ((((((((((((((((((((((((((Immunotherapy[MeSH Terms]) OR (Immunotherapies[Title/Abstract])) OR (Immunotherapy[Title/Abstract])) OR ("immune checkpoint inhibitor*"[Title/Abstract])) OR ("immune checkpoint blockade"[Title/Abstract])) OR ("immune therapy"[Title/Abstract])) OR ("immunotherapeutic agents"[Title/Abstract])) OR ("cancer immunotherapy"[Title/Abstract])) OR ("tumor immunotherapy"[Title/Abstract])) OR ("PD-1 inhibitor"[Title/Abstract])) OR ("anti-PD-1"[Title/Abstract])) OR ("programmed cell death protein 1 inhibitor"[Title/Abstract])) OR (pembrolizumab[Title/Abstract])) OR (nivolumab[Title/Abstract])) OR ("PD-L1 inhibitor"[Title/Abstract])) OR ("anti-PD-L1"[Title/Abstract])) OR ("programmed death ligand 1 inhibitor"[Title/Abstract])) OR (atezolizumab[Title/Abstract])) OR (durvalumab[Title/Abstract])) OR ("CTLA-4 inhibitor"[Title/Abstract])) OR ("anti-CTLA-4"[Title/Abstract])) OR ("dual checkpoint blockade"[Title/Abstract])) OR ("PD-1 + CTLA-4 combination"[Title/Abstract])) OR (chemoimmunotherapy[Title/Abstract])) OR ("chemotherapy plus immunotherapy"[Title/Abstract])) AND (((((((((((Ovarian Neoplasms[MeSH Terms]) OR ("Ovarian Neoplasm"[Title/Abstract])) OR ("Ovary Neoplasms"[Title/Abstract])) OR ("Ovary Neoplasm"[Title/Abstract])) OR ("Ovary Cancer"[Title/Abstract])) OR ("Ovary Cancers"[Title/Abstract])) OR ("Cancer of Ovary"[Title/Abstract])) OR ("Ovarian Cancer"[Title/Abstract])) OR ("Ovarian Cancers"[Title/Abstract])) OR ("carcinoma of ovary"[Title/Abstract])) OR ("ovary carcinoma"[Title/Abstract]))) AND ((randomized controlled trial[pt] OR controlled clinical trial[pt] OR randomized[tiab] OR placebo[tiab] OR drug therapy[sh] OR randomly[tiab] OR trial[tiab] OR groups[tiab]) NOT (animals[mh] NOT humans[mh])) |
| --- | --- |
| #7 | Search: (randomized controlled trial[pt] OR controlled clinical trial[pt] OR randomized[tiab] OR placebo[tiab] OR drug therapy[sh] OR randomly[tiab] OR trial[tiab] OR groups[tiab]) NOT (animals[mh] NOT humans[mh]) |
| #6 | Search: ((((((((((((((((((((((((Immunotherapy[MeSH Terms]) OR (Immunotherapies[Title/Abstract])) OR (Immunotherapy[Title/Abstract])) OR ("immune checkpoint inhibitor*"[Title/Abstract])) OR ("immune checkpoint blockade"[Title/Abstract])) OR ("immune therapy"[Title/Abstract])) OR ("immunotherapeutic agents"[Title/Abstract])) OR ("cancer immunotherapy"[Title/Abstract])) OR ("tumor immunotherapy"[Title/Abstract])) OR ("PD-1 inhibitor"[Title/Abstract])) OR ("anti-PD-1"[Title/Abstract])) OR ("programmed cell death protein 1 inhibitor"[Title/Abstract])) OR (pembrolizumab[Title/Abstract])) OR (nivolumab[Title/Abstract])) OR ("PD-L1 inhibitor"[Title/Abstract])) OR ("anti-PD-L1"[Title/Abstract])) OR ("programmed death ligand 1 inhibitor"[Title/Abstract])) OR (atezolizumab[Title/Abstract])) OR (durvalumab[Title/Abstract])) OR ("CTLA-4 inhibitor"[Title/Abstract])) OR ("anti-CTLA-4"[Title/Abstract])) OR ("dual checkpoint blockade"[Title/Abstract])) OR ("PD-1 + CTLA-4 combination"[Title/Abstract])) OR (chemoimmunotherapy[Title/Abstract])) OR ("chemotherapy plus immunotherapy"[Title/Abstract]) |
| #5 | Search: (Immunotherapies[Title/Abstract])) OR (Immunotherapy[Title/Abstract])) OR ("immune checkpoint inhibitor*"[Title/Abstract])) OR ("immune checkpoint blockade"[Title/Abstract])) OR ("immune therapy"[Title/Abstract])) OR ("immunotherapeutic agents"[Title/Abstract])) OR ("cancer immunotherapy"[Title/Abstract])) OR ("tumor immunotherapy"[Title/Abstract])) OR ("PD-1 inhibitor"[Title/Abstract])) OR ("anti-PD-1"[Title/Abstract])) OR ("programmed cell death protein 1 inhibitor"[Title/Abstract])) OR (pembrolizumab[Title/Abstract])) OR (nivolumab[Title/Abstract])) OR ("PD-L1 inhibitor"[Title/Abstract])) OR ("anti-PD-L1"[Title/Abstract])) OR ("programmed death ligand 1 inhibitor"[Title/Abstract])) OR (atezolizumab[Title/Abstract])) OR (durvalumab[Title/Abstract])) OR ("CTLA-4 inhibitor"[Title/Abstract])) OR ("anti-CTLA-4"[Title/Abstract])) OR ("dual checkpoint blockade"[Title/Abstract])) OR ("PD-1 + CTLA-4 combination"[Title/Abstract])) OR (chemoimmunotherapy[Title/Abstract])) OR ("chemotherapy plus immunotherapy"[Title/Abstract]) |
| #4 | Search: Immunotherapy[MeSH Terms] |
| #3 | Search: ((((((((((Ovarian Neoplasms[MeSH Terms]) OR ("Ovarian Neoplasm"[Title/Abstract])) OR ("Ovary Neoplasms"[Title/Abstract])) OR ("Ovary Neoplasm"[Title/Abstract])) OR ("Ovary Cancer"[Title/Abstract])) OR ("Ovary Cancers"[Title/Abstract])) OR ("Cancer of Ovary"[Title/Abstract])) OR ("Ovarian Cancer"[Title/Abstract])) OR ("Ovarian Cancers"[Title/Abstract])) OR ("carcinoma of ovary"[Title/Abstract])) OR ("ovary carcinoma"[Title/Abstract]) |
| #2 | Search: ("Ovarian Neoplasm"[Title/Abstract])) OR ("Ovary Neoplasms"[Title/Abstract])) OR ("Ovary Neoplasm"[Title/Abstract])) OR ("Ovary Cancer"[Title/Abstract])) OR ("Ovary Cancers"[Title/Abstract])) OR ("Cancer of Ovary"[Title/Abstract])) OR ("Ovarian Cancer"[Title/Abstract])) OR ("Ovarian Cancers"[Title/Abstract])) OR ("carcinoma of ovary"[Title/Abstract])) OR ("ovary carcinoma"[Title/Abstract]) |
| #1 | Search: Ovarian Neoplasms[MeSH Terms] |

# Supplementary 2: Characteristics of studies and subjects included in the review

| **Study** | **Study Design** | **Country/Region** | **Subjects  (intervention/ control)** | **Mean age  (intervention/ control)** | **Disease type** | **Intervention detail** | | **Treatment cycle** | **Outcomes** |
| --- | --- | --- | --- | --- | --- | --- | --- | --- | --- |
|  |  |  |  |  |  | **Intervention group** | **Control group** |  |  |
| Brewer et al. (2019) | Open-label RCT | Italy & USA | 97 (47/50) | 57.4 ± 11.36 vs. 57.5 ± 10.92 | FIGO stage III/IV epithelial ovarian cancer, surgically debulked to ≤1 cm | Oregovomab 2 mg IV was administered after the 1st, 3rd, and 5th cycles of chemotherapy, and once more 12 weeks after the end of chemotherapy (4 times in total) | IV paclitaxel 175 mg/m² + carboplatin AUC 6, 1 cycle every 3 weeks, for a total of 6 cycles | 18 weeks (6 cycles × 3 weeks) + single immune maintenance administration at week 19–30 | OS, PFS, TRAEs |
| Buzzonetti et al. (2014) | Double-blind RCT | Italy | 129 (91/38) | NA | Epithelial ovarian cancer in first complete clinical remission | Abagovomab 20 mg sc q2w × 3 induction, q4w maintenance, up to 21 months | Placebo sc dosing schedule is exactly the same (q2w × 3, q4w maintenance) | 6 weeks induction phase (every 2 weeks for 3 times), then every 4 weeks for up to 21 months | OS, PFS |
| Alberts et al. (1989) | Open-label RCT | USA | 128 (65/63) | 58.6±9.5 vs. 55.5±9.4 | FIGO stage III/IV epithelial ovarian cancer, residual size ≥ 2 cm after surgery | DC+BCG: Doxorubicin 40 mg/m² IV D1 + Cyclophosphamide 600 mg/m² IV D1 + BCG Connaught scarification D8 & D15 + Cisplatin 50 mg/m² IV D1 | Doxorubicin 40 mg/m² IV D1 + Cyclophosphamide 600 mg/m² IV D1 + Cisplatin 50 mg/m² IV D1 | 1 cycle every 3–4 weeks for 12 cycles | OS, PFS, ORR |
| González-Martín et al. (2024) | Double-blind RCT | Multicenter | 417 (208/209) | 63 ± 16.5 vs. 62 ± 18.8 | Measurable high-grade serous, endometrioid, or undifferentiated recurrent ovarian cancer with TFIp > 6 months | Induction: Atezolizumab 1,200 mg IV D1 q21d (or 840 mg IV D1 & 15 q28d per CT backbone) + investigator-chosen carboplatin doublet × 6 cycles Maintenance: Atezolizumab same dosing + Niraparib daily (300 mg or 200 mg if weight < 77 kg/platelets < 150×10^9/L) until progression | Induction: Placebo IV (matched schedule) + same carboplatin doublet × 6 cycles Maintenance: Placebo same schedule + Niraparib daily (300 mg or 200 mg) until progression | 6 cycles CT induction (18 weeks), maintenance until progression (median follow-up 28.6 months) | OS, PFS, ORR, DCR, TRAEs |
| Barlow et al. (1980) | Open-label RCT | USA | 46 (20/26) | 56.6 ± 11.1 vs 56.8 ± 10.2 | FIGO stage III–IV epithelial ovarian adenocarcinoma | C. parvum 2 mg/m² IV infusion: Starting on day 6 (1 day after chemotherapy), once every 4 weeks for 12 times; then 4 times every 8 weeks, and then every 12 weeks until progression. Chemotherapy regimen: MECY or FUCY | Chemotherapy regimen (MECY or FUCY) without C. parvum administration. | Chemotherapy is given in cycles of 4 weeks, for a maximum of 6 cycles; immunity can be maintained for up to 24 months. | ORR |
| Berek et al. (2008) | Double-blind RCT | USA | 371 (251/120) | 58.8 ± 10.44 vs 59.6 ± 10.59 | Stage III–IV epithelial ovarian cancer, residual size ≤ 2 cm after surgery, CA-125 normalized | Oregovomab 2 mg IV over 20 min at weeks 0, 4, 8 then q12w until relapse or 5 y | Placebo IV (matched) same schedule | Induction (wk 0–8), then maintenance q12w (median follow-up 29 mo) | OS, PFS |
| Moore et al. (2021) | Double-blind RCT | Multicenter | 1301 (651/650) | 58.3 ± 13.8 vs. 54.8 ± 16.3 | Measurable or macroscopic residual epithelial ovarian, fallopian tube, or primary peritoneal cancer, FIGO Ⅲ–Ⅳ | Atezolizumab 1,200 mg IV D1 q21d (cycles 1–22); Paclitaxel 175 mg/m² + Carboplatin AUC 6 IV D1 (cycles 1–6); Bevacizumab 15 mg/kg IV D1 (cycles 2–22; paused during interval surgery in neoadjuvant patients) | Same dose of placebo IV q21d + same chemotherapy + same Bevacizumab dosing regimen | Chemotherapy induction for 6 cycles (18 weeks), immune/placebo + Bev maintenance until progression (median follow-up 19.9 months) | PFS, ORR, DCR, TRAEs |
| Hamanishi et al. (2021) | Open-label RCT | Japan | 316 (157/159) | 57.3 ± 13.8 vs. 58.5 ± 11.5 | Platinum-resistant epithelial ovarian cancer (incl. fallopian-tube & primary peritoneal carcinoma) | Nivolumab 240 mg IV q2 weeks (one cycle) until progression | Investigator’s choice of chemotherapy: Gemcitabine 1,000 mg/m² IV D1, 8, 15 q28 days; or Pegylated liposomal doxorubicin 50 mg/m² IV q28 days | Nivolumab cycles every 2 weeks; chemotherapy cycles defined above; until progression or unacceptable toxicity | OS, PFS, ORR, DCR, TRAEs |
| Kurtz et al. (2023) | Double-blind RCT | Multicenter | 614 (410/204) | 58.0 ± 10.4 vs. 59.0 ± 12.6 | Recurrent platinum-sensitive epithelial ovarian, fallopian-tube, or primary peritoneal carcinoma (PFI > 6 months) | Induction: Atezolizumab 1,200 mg IV D1 q21d (or 840 mg IV D1&D15 q28d if per chemo backbone) + investigator-chosen platinum doublet × 6 cycles + concurrent bevacizumab (15 mg/kg)  Maintenance: Atezolizumab same dosing + bevacizumab 15 mg/kg q21d up to 24 months or until progression | Placebo IV matched schedule + same chemotherapy doublet × 6 + bevacizumab induction/maintenance as above | 6 cycles chemotherapy (~18 weeks) + maintenance atezolizumab/placebo + bevacizumab until progression (median follow-up 36.6 months) | OS, PFS, ORR, DCR, TRAEs |
| Rob et al. (2022) | Open-label RCT | Czech Republic & Poland | 61 (31/30) | 61.7 ± 12.1 vs. 62.3 ± 7.0 | FIGO III EOC (serous/endometrioid/mucinous) | DCVAC/OvCa ~10×10⁶ DCs sc，parallel to CT (paclitaxel 175 mg/m² + carboplatin AUC 5–7 q3 w ×6) | Paclitaxel 175 mg/m² + carboplatin AUC 5–7 q3 w ×6 alone | 6 cycles CT (18 wk); DCVAC/OvCa dosing over 9.7 mo | PFS, TRAEs |
| Kristeleit et al. (2017) | Open-label RCT | Multicenter | 42 (22/20) | 59.0 ± 13.8 vs. 60.3 ± 8.5 | FIGO stage I–IV epithelial ovarian, primary peritoneal, or fallopian tube cancer with recurrent CA-125 | Epacadostat 600 mg PO BID, 28 days/cycle until disease progression or discontinuation | Tamoxifen 20 mg PO BID, 28 days/cycle, same discontinuation criteria | 28 days/cycle, until progression or intolerance | PFS, ORR, DCR, TRAEs |
| Lee et al. (2022) | Open-label RCT | Multicenter | 32 (16/16) | 61.2 ± 10.5 vs. 60.8 ± 11.0 | Platinum-sensitive recurrent epithelial ovarian cancer, HRD-positive | Olaparib 300 mg PO BID + Cediranib 30 mg PO daily, 28-day cycle | Olaparib 300 mg PO BID + Durvalumab 1,500 mg IV q4w, 28-day cycle | 28-day cycle, until disease progression or intolerance | OS, PFS, ORR, DCR, TRAEs |
| Monk et al. (2017) | Double-blind RCT | USA | 294 (147/147) | 61.0 ± 15.4 vs. 62.9 ± 11.3 | Recurrent or persistent epithelial ovarian carcinoma (incl. fallopian tube & primary peritoneal carcinoma) | PLD 40 mg/m² IV D1 q28d + motolimod 3.0 mg/m² SC D3,10,17 q28d (cycles 1–4), then D3 q28d until progression | PLD 40 mg/m² IV D1 q28d + placebo SC D3,10,17 q28d (same schedule) | 28-day cycle until disease progression | OS, PFS, TRAEs |
| Hinchcliff et al. (2024) | Open-label RCT | USA | 61 (38/23) | 60.0 ± 11.3 vs. 61.0 ± 11.3 | Platinum-resistant high-grade serous ovarian carcinoma | Tremelimumab 1 mg/kg IV + Durvalumab 1.5 g IV on Day 1 of each 28-day cycle for 4 cycles, followed by Durvalumab 1.5 g IV every 4 weeks (up to 9 additional doses). | Tremelimumab 3 mg/kg IV on Day 1 of each 28-day cycle for 4 cycles; upon disease progression, switch to Durvalumab 1.5 g IV every 4 weeks (up to 9 doses). | 28-day cycles; up to 4 doses ICI combination/induction, followed by up to 9 doses durvalumab maintenance, or switch at sequential clinical progression | OS, PFS, ORR, DCR, TRAEs |
| Oei et al. (2008) | Open-label RCT | Netherlands | 447 (224/223) | 54.0 ± 13.8 vs. 52.0 ± 14.0 | FIGO Ic–IV epithelial ovarian carcinoma in complete remission after surgery + platinum chemo, minimal/no residual disease at second-look | Single IP injection of ⁹⁰Y-muHMFG1 at 666 MBq/m² (max 1,110 MBq/m²) plus 20 mg unlabeled HMFG1, administered 4–8 weeks post-chemotherapy | Standard follow-up (no IP antibody) | Single IP dose; all other post-chemo care per standard | OS, PFS |
| Oh et al. (2016) | Open-label RCT | USA | 42 (31/11) | 62 ± 11 vs. 57 ± 10.5 | FIGO stage III–IV epithelial ovarian cancer (including fallopian tube cancer and primary peritoneal cancer) | Vigil® 1.0×10⁷ cells/time, intradermal injection, once a month, for 4–12 times | Standard-of-care observation (no maintenance treatment) | 1 28-day cycle per month until vaccine is exhausted or relapse occurs; up to 12 months | PFS |
| Kim et al. (2023) | Open-label RCT | South Korea | 30 (16/14) | 57.6 ± 10.1 vs. 59.2 ± 9.8 | Platinum-resistant recurrent epithelial ovarian cancer (including fallopian tube/primary peritoneal cancer) with HRR gene mutations | Olaparib 200 mg PO bid + Cediranib 30 mg PO qd, 28 days/cycle until progression | Olaparib 300 mg PO bid + Durvalumab 1,500 mg IV q4w (from cycle 2) until progression or 12 months | 28 days/cycle, continued until disease progression | PFS, ORR, DCR, TRAEs |
| Ray-Coquard et al. (2024) | Open-label RCT | France | 91 (61/30) | NA | FIGO IIIC/IV high-grade serous carcinoma (including ovarian, fallopian tube, and primary peritoneal cancer) | Pembrolizumab 200 mg q3w + Carboplatin AUC 5–6 q3w + Paclitaxel 175 mg/m² q3w | Carboplatin AUC 5–6 q3w + Paclitaxel 175 mg/m² q3w | 4 preoperative cycles + 2–5 postoperative cycles, pembrolizumab administered for up to 2 years | OS, PFS, ORR, DCR, TRAEs |
| Rocconi et al. (2020) | Double-blind RCT | USA | 91 (47/44) | 63.0 ± 10.5 | Stage III/IV high-grade serous, endometrioid, or clear-cell epithelial ovarian cancer | Gemogenovatucel-T 1×10⁷ cells intradermal × monthly for 4–12 doses | Placebo intradermal × monthly, identical schedule | 1 injection per 28-day cycle, minimum 4 and up to 12 cycles | OS, PFS, TRAEs |
| Rocconi et al. (2021) | Open-label RCT | USA | 21 (11/10) | 63.9 ± 6.3 vs. 59.7 ± 8.3 | Relapsed high-grade epithelial ovarian cancer | Vigil: 1 × 10⁶–10⁷ cells/mL i.d. q21d, 4–12 cycles | Atezo: 1200 mg i.v. q21d, 2 cycles, Vigil+i.v. thereafter | 1 dose every 28 days, minimum 4, maximum 12 cycles | OS, PFS, TRAEs |
| Pujade-Lauraine et al. (2021) | Open-label RCT | Multicenter | 376 (188/188) | 60.0 ± 10.4 vs. 61.0 ± 12.2 | Platinum-resistant or platinum-refractory epithelial ovarian, fallopian-tube, or primary peritoneal carcinoma | Avelumab: 10 mg/kg IV over 1 h every 2 weeks | PLD 40 mg/m² IV q4w alone, with identical premedication | Until RECIST-confirmed progression, unacceptable toxicity, or withdrawal | OS, PFS, ORR, DCR, TRAEs |
| Monk et al. (2021) | Open-label RCT | Multicenter | 666 (331/335) | 60.0 ± 11.9 vs. 61.0 ± 12.2 | Stage III–IV epithelial ovarian, fallopian-tube or primary peritoneal carcinoma | Avelumab 10 mg/kg IV q3w concurrently with chemotherapy as above (6 cycles), then Avelumab 10 mg/kg IV q2w until progression or up to 24 months | Carboplatin + Paclitaxel same dose regimen (6 cycles), followed by observation only | 6 cycles of chemotherapy; maintenance period 24 months or until progression | OS, PFS, ORR, TRAEs |
| Banerjee et al. (2024) | Open-label RCT | Multicenter | 62 (31/31) | 63.0 ± 10.0 vs. 64.0 ± 9.8 | FIGO IIIC-IV platinum-resistant epithelial ovarian cancer (including fallopian tube/primary peritoneal) | Bevacizumab 15 mg/kg IV q3w + Atezolizumab 1 200 mg IV q3w + placebo daily | Bevacizumab 15 mg/kg IV q3w alone | Until progression or intolerance confirmed by RECIST v1.1 | OS, PFS, ORR, DCR, TRAEs |
| Mutch et al. (2024) | Open-label RCT | Multicenter | 76 (39/37) | 62.8 ± 9.3 vs. 65.3 ± 8.8 | Platinum-sensitive recurrent ovarian cancer (high-grade serous and grade 2/3 endometrioid) | Cobimetinib 60 mg PO qd days 1–21 + Niraparib 200 mg PO qd days 1–28 + Atezolizumab 840 mg IV days 1 & 15; 28-day cycles | Cobimetinib 60 mg PO qd days 1–21 + Niraparib 200 mg PO qd days 1–28; 28-day cycles | 28 days per cycle, continued until disease progression or unacceptable toxicity | PFS, ORR, TRAEs |
| Cibula et al. (2021) | Open-label RCT | Multicenter | 71 (39/32) | 59.8 ± 9.5 vs. 59.5 ± 7.8 | Platinum-sensitive recurrent epithelial ovarian cancer (Serous 93.8% vs 90.6%; Endometrioid, Mucinous) | DCVAC/OvCa: Autologous dendritic cell vaccine, 10 doses (first 5 doses every 3 weeks, next 5 doses every 6 weeks), 2×2.5 mL subcutaneously; Chemotherapy: Carboplatin AUC 4–5 IV d1 + Gemcitabine 1000 mg/m² IV d1,8, every 21 days for 1 cycle | Carboplatin AUC 4–5 IV d1 + Gemcitabine 1000 mg/m² IV d1,8, every 21 days as a cycle | 6–10 21-day cycles; DCVAC/OvCa begins after the second chemotherapy cycle until 10 doses or disease progression | OS, PFS, ORR, TRAEs |
| Zamarin et al. (2020) | Open-label RCT | USA | 100 (49/51) | 62.5 ± 12.5 vs. 63.5 ± 13.5 | Recurrent or persistent epithelial ovarian cancer | Nivolumab 3 mg/kg IV q2 weeks × 4 doses (induction) → maintenance nivolumab 3 mg/kg IV q2 weeks up to 42 doses | Nivolumab 3 mg/kg IV q2 weeks + Ipilimumab 1 mg/kg IV q3 weeks × 4 doses (induction) → maintenance nivolumab 3 mg/kg IV q2 weeks up to 42 doses | Induction phase: 4 doses (administered at weeks 1, 3, 5, and 7), followed by maintenance therapy every 2 weeks until 42 doses or discontinuation due to progression/toxicity | OS, PFS, ORR, DCR, TRAEs |

Note: RCT, randomized controlled trial; PD-1, programmed death-1; PD-L1, programmed death-ligand 1; CTLA-4, cytotoxic T-lymphocyte-associated antigen 4; ICI, immune checkpoint inhibitor; mAb, monoclonal antibody; q2W (q2w), every 2 weeks; q3W (q3w), every 3 weeks; q4W (q4w), every 4 weeks; q12W (q12w), every 12 weeks; IV, intravenous; PO, per os (oral); sc, subcutaneous; BID, bis in die (twice daily); AUC, area under the curve; PLD, pegylated liposomal doxorubicin; OS, overall survival; PFS, progression-free survival; ORR, objective response rate; DCR, disease control rate; TRAEs, treatment-related adverse events; AE, adverse event; CON, control group; m, months; w, weeks; NA, not available.

# Supplementary 3: Risk of Bias

| **Author** | **Bias arising from the randomization process** | **Bias due to deviations from intended intervention** | **Bias due to missing outcome data** | **Bias in measurement of the outcome** | **Bias in selection of the reported result** | **Overall** |
| --- | --- | --- | --- | --- | --- | --- |
| Brewer et al. (2019) | Low | Low | Low | Low | Low | Low |
| Buzzonetti et al. (2014) | Low | Low | Low | Low | Low | Low |
| Alberts et al. (1989) | Low | Low | Low | Some Concerns | Low | Some Concerns |
| González-Martín et al. (2024) | Some Concerns | Low | Low | Low | Low | Some Concerns |
| Barlow et al. (1980) | Low | Low | Low | Low | Low | Low |
| Berek et al. (2008) | Low | Low | Low | Low | Low | Low |
| Moore et al. (2021) | Low | Low | Low | Low | Low | Low |
| Hamanishi et al. (2021) | Low | Low | Some Concerns | Low | Low | Some Concerns |
| Kurtz et al. (2023) | Some Concerns | Low | Some Concerns | Low | Low | Some Concerns |
| Rob et al. (2022) | Low | Low | Low | Low | Low | Low |
| Kristeleit et al. (2017) | Low | Low | Low | Low | Low | Low |
| Lee et al. (2022) | Some Concerns | Low | Low | Low | Low | Some Concerns |
| Monk et al. (2017) | Low | Low | Low | Low | Low | Low |
| Hinchcliff et al. (2024) | High | High | High | Low | Low | High |
| Oei et al. (2008) | Low | Low | Low | Low | Low | Low |
| Oh et al. (2016) | Low | Low | Some Concerns | Low | Low | Some Concerns |
| Kim et al. (2023) | Some Concerns | Low | Some Concerns | Low | Low | Some Concerns |
| Ray-Coquard et al. (2024) | Low | High | High | Low | Low | High |
| Rocconi et al. (2020) | Low | Low | Low | Low | Low | Low |
| Rocconi et al. (2021) | Some Concerns | Some Concerns | Some Concerns | Low | Low | Some Concerns |
| Pujade-Lauraine et al. (2021) | Low | Some Concerns | Low | Low | Low | Some Concerns |
| Monk et al. (2021) | Low | Low | Low | Low | Low | Low |
| Banerjee et al. (2024) | Some Concerns | Low | Low | Low | Low | Some Concerns |
| Mutch et al. (2024) | High | Some Concerns | Some Concerns | Low | Low | High |
| Cibula et al. (2021) | Low | Low | Some Concerns | Low | Low | Some Concerns |
| Zamarin et al. (2020) | Low | Low | Low | Low | Low | Low |

# Supplementary 4: Funnel Plots of Publication Bias


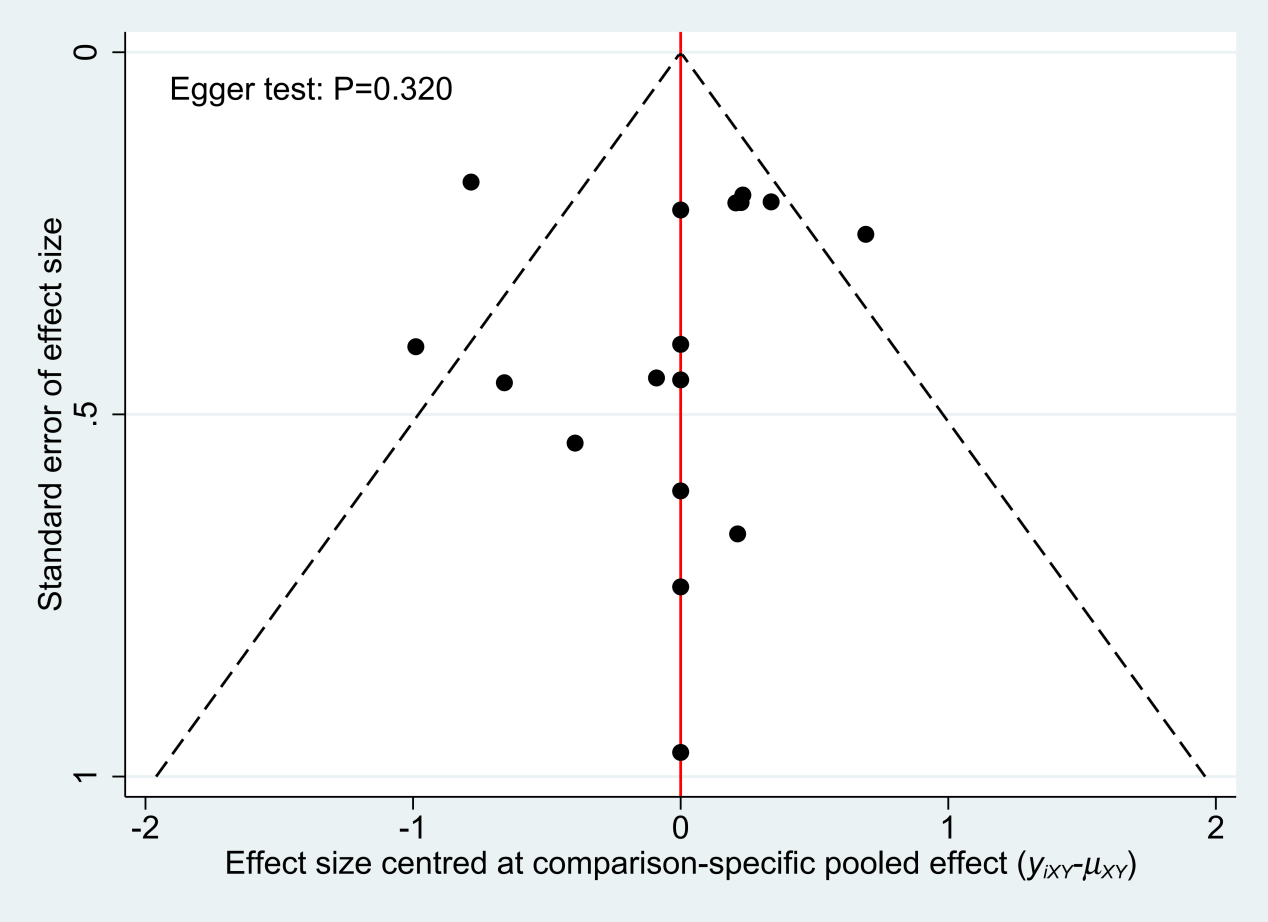


Figure 4.1 Overall survival (Egger’s test: p = 0.320).


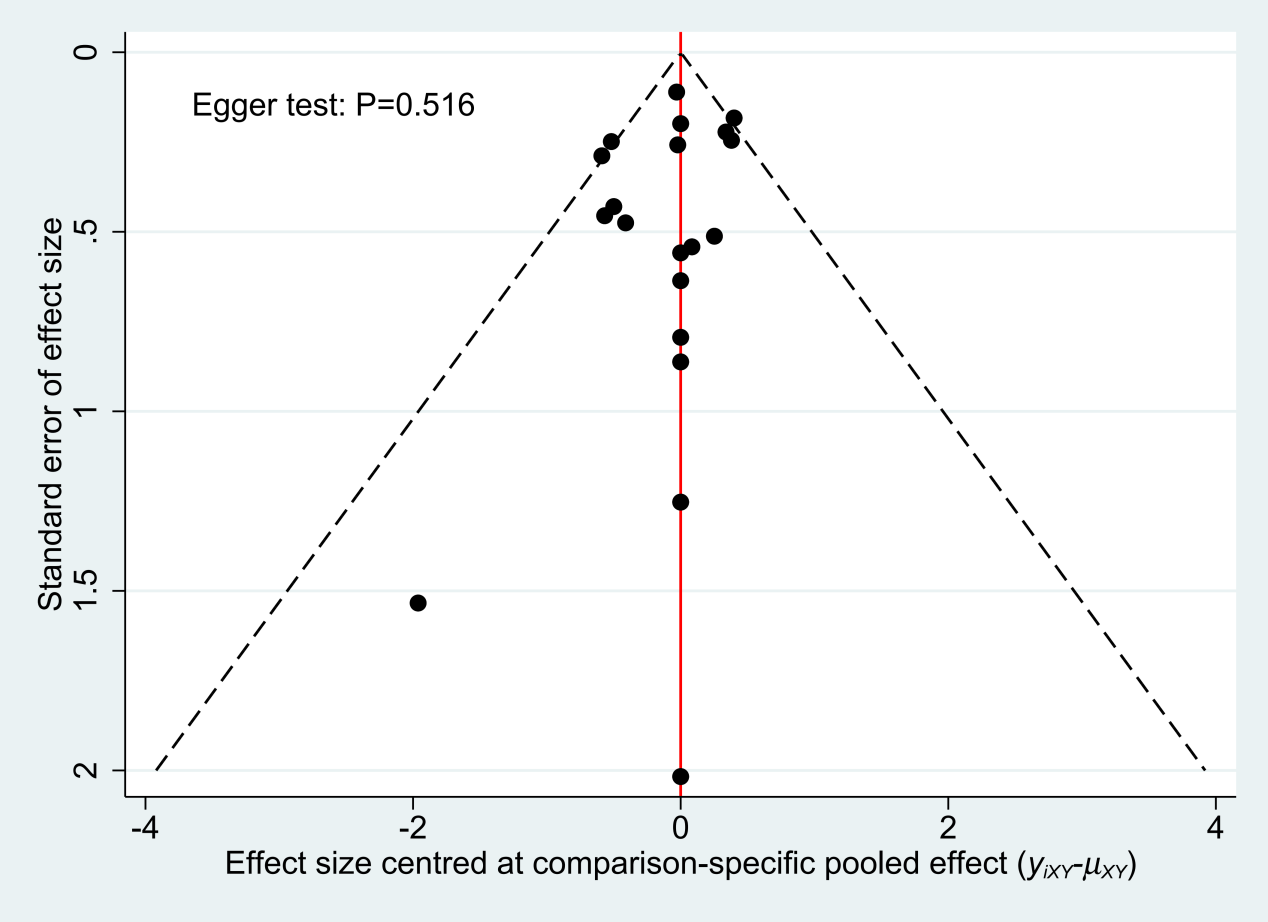


Figure 4.2 Progression-free survival (Egger’s test: p = 0.516).


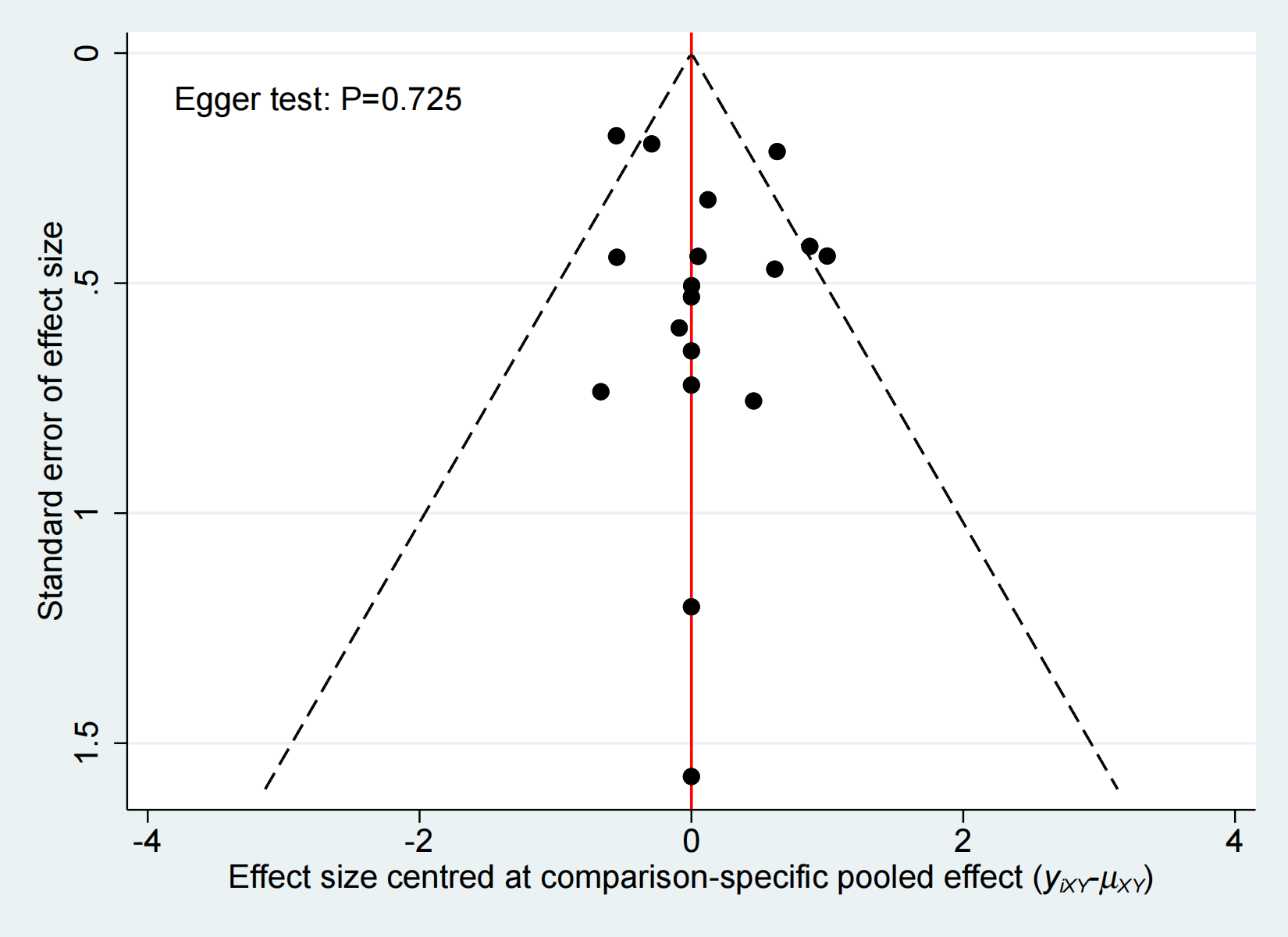


Figure 4.3 Objective response rate (Egger’s test: p = 0.725).


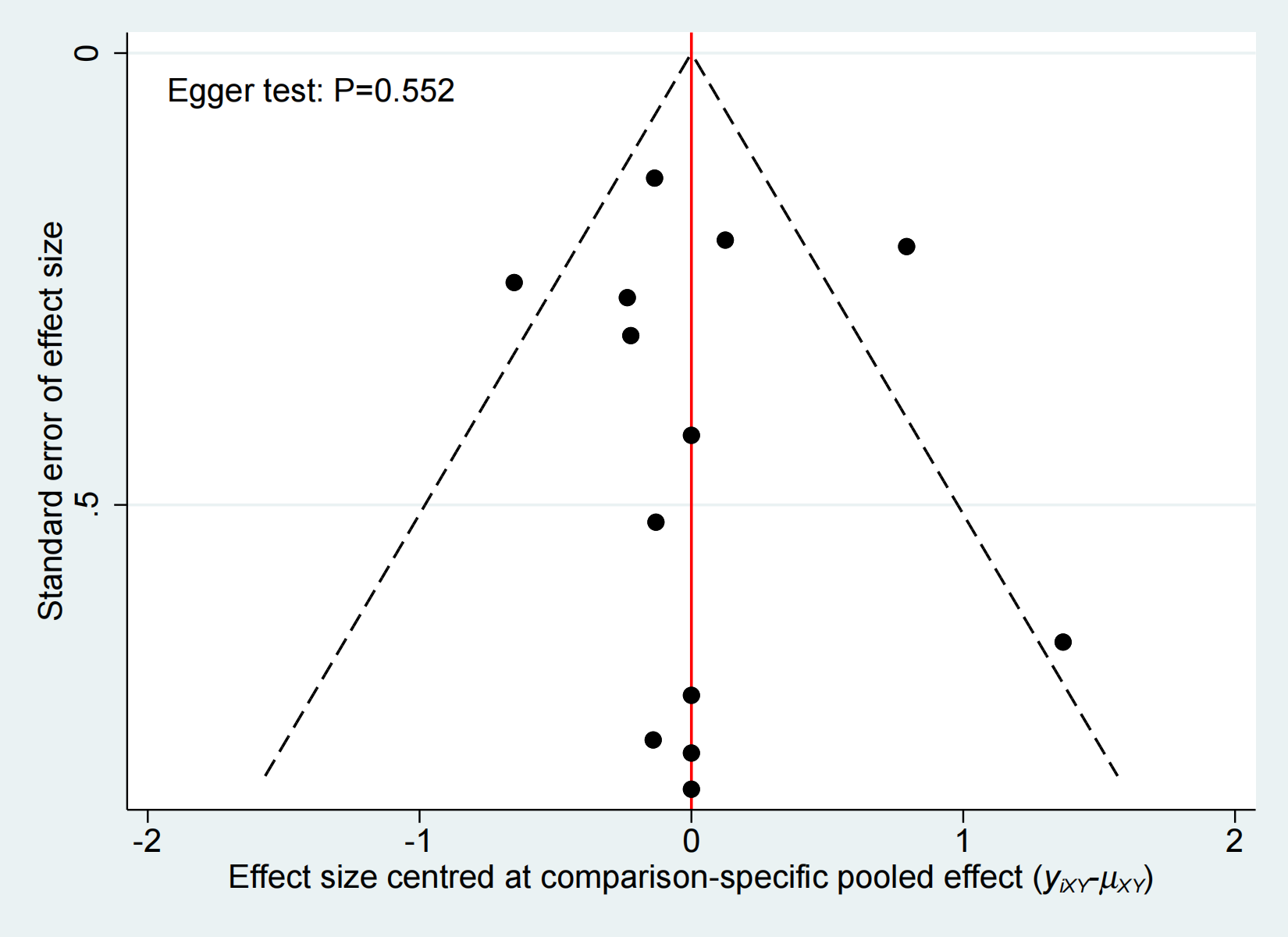


Figure 4.4. Disease control rate (Egger’s test: p = 0.552).


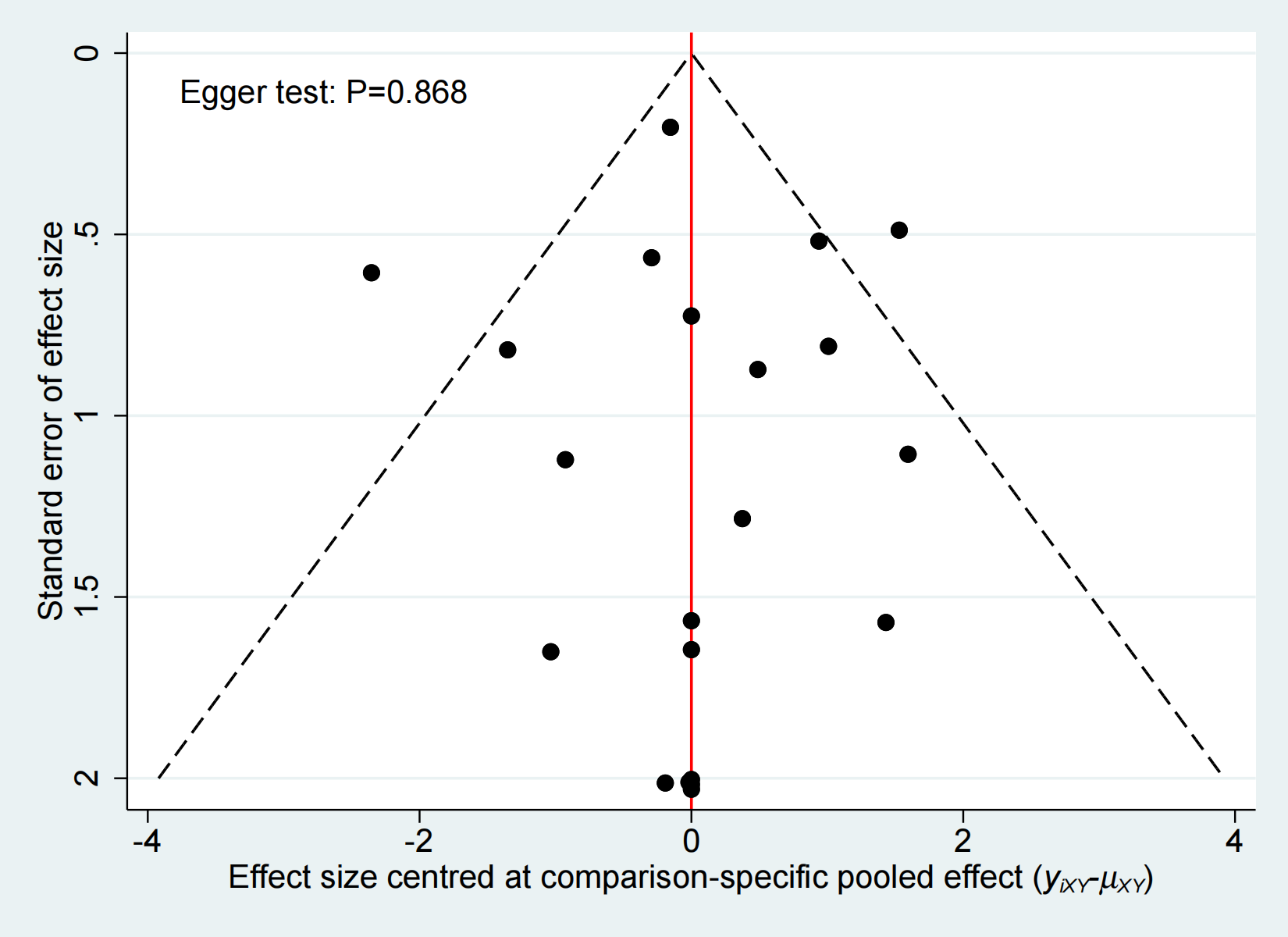


Figure 4.5. Treatment-related adverse events (Egger’s test: p = 0.868).


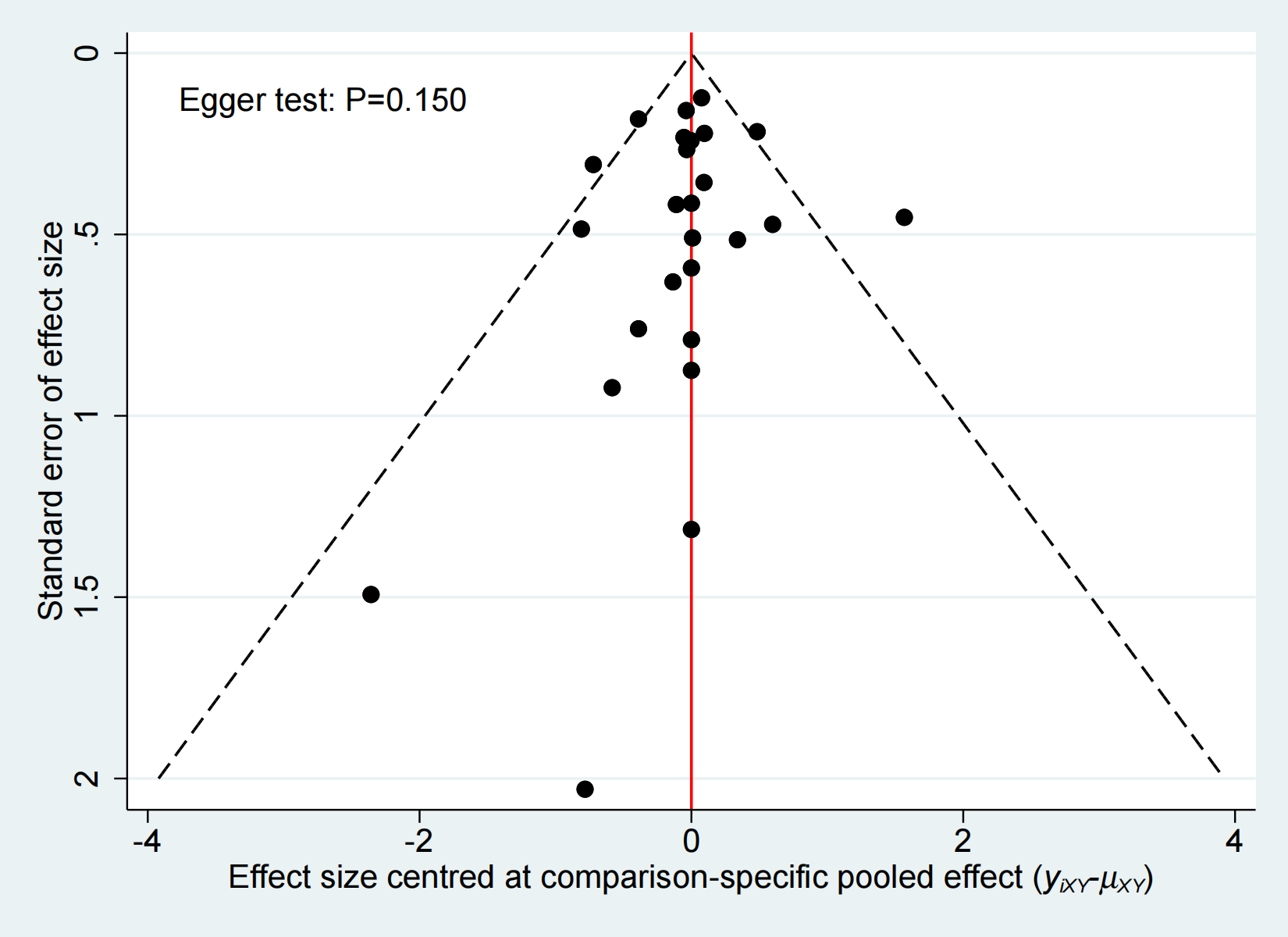


Figure 4.6. Grade ≥ 3 treatment-related adverse events (Egger’s test: p = 0.150).

# Supplementary 5: Forest plot


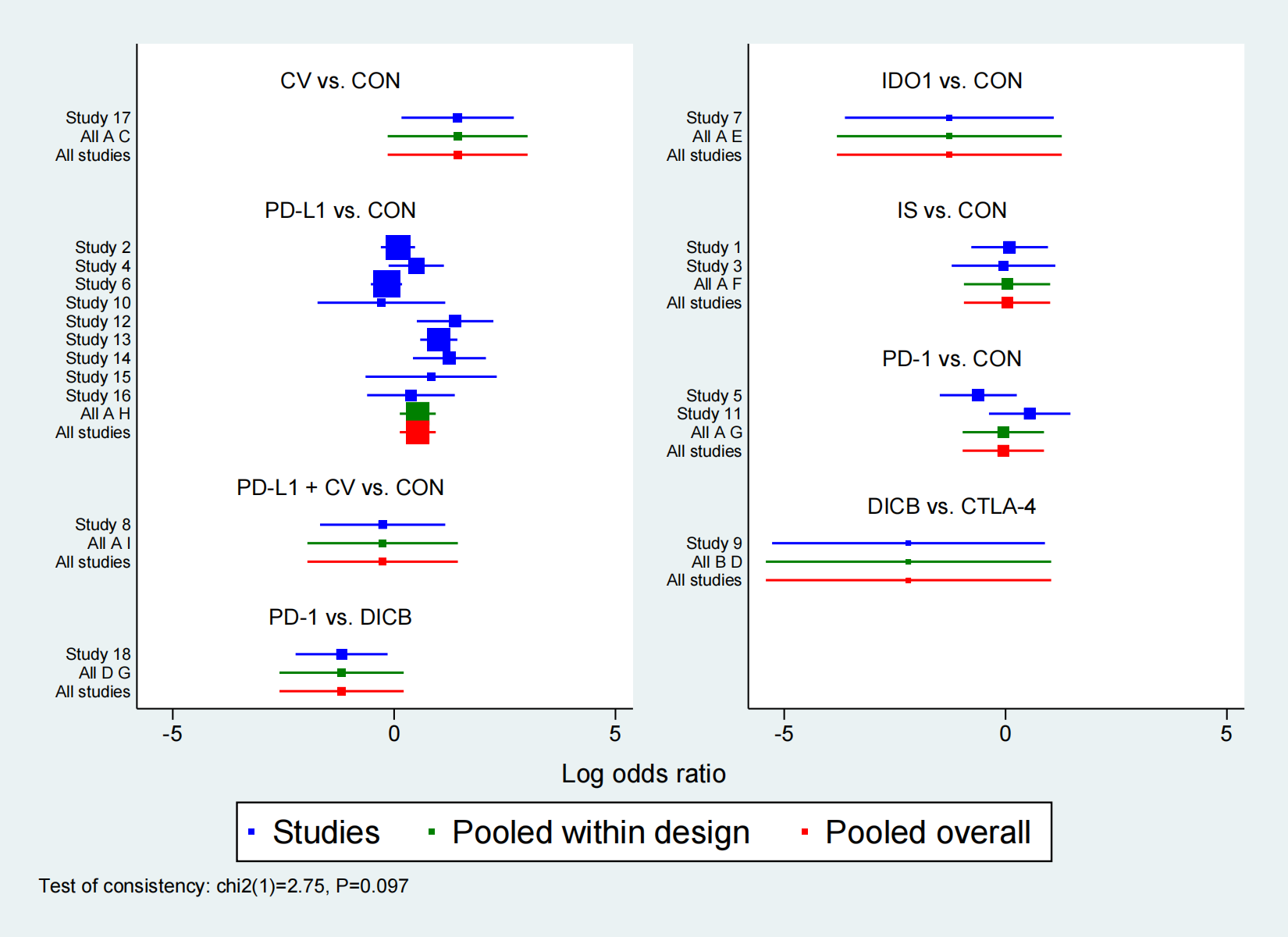


Figure 5.1. Forest plot of objective response rate (Consistency’s test: p = 0.097).


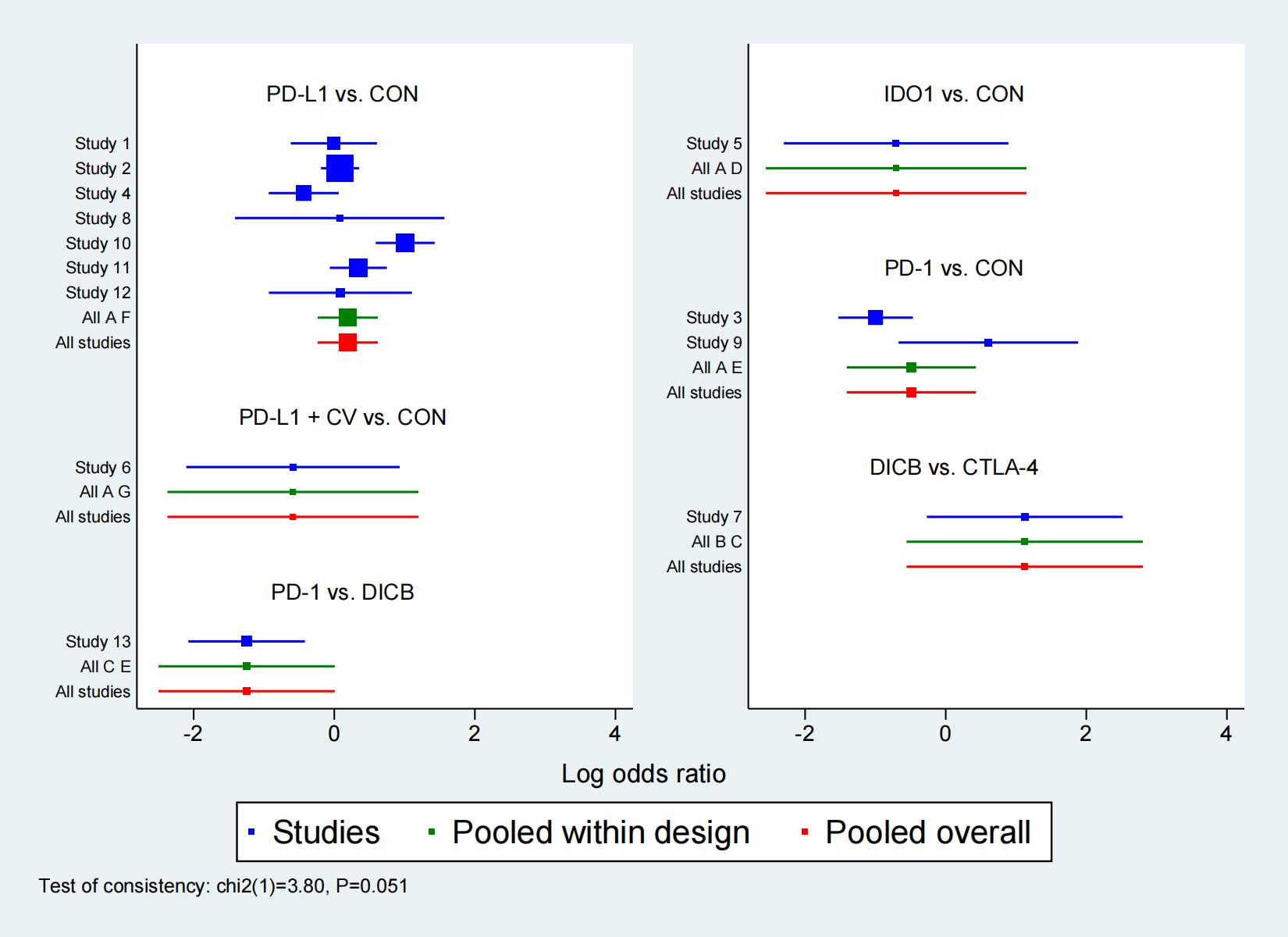


Figure 5.2. Forest plot of disease control rate (Consistency’s test: p = 0.051).


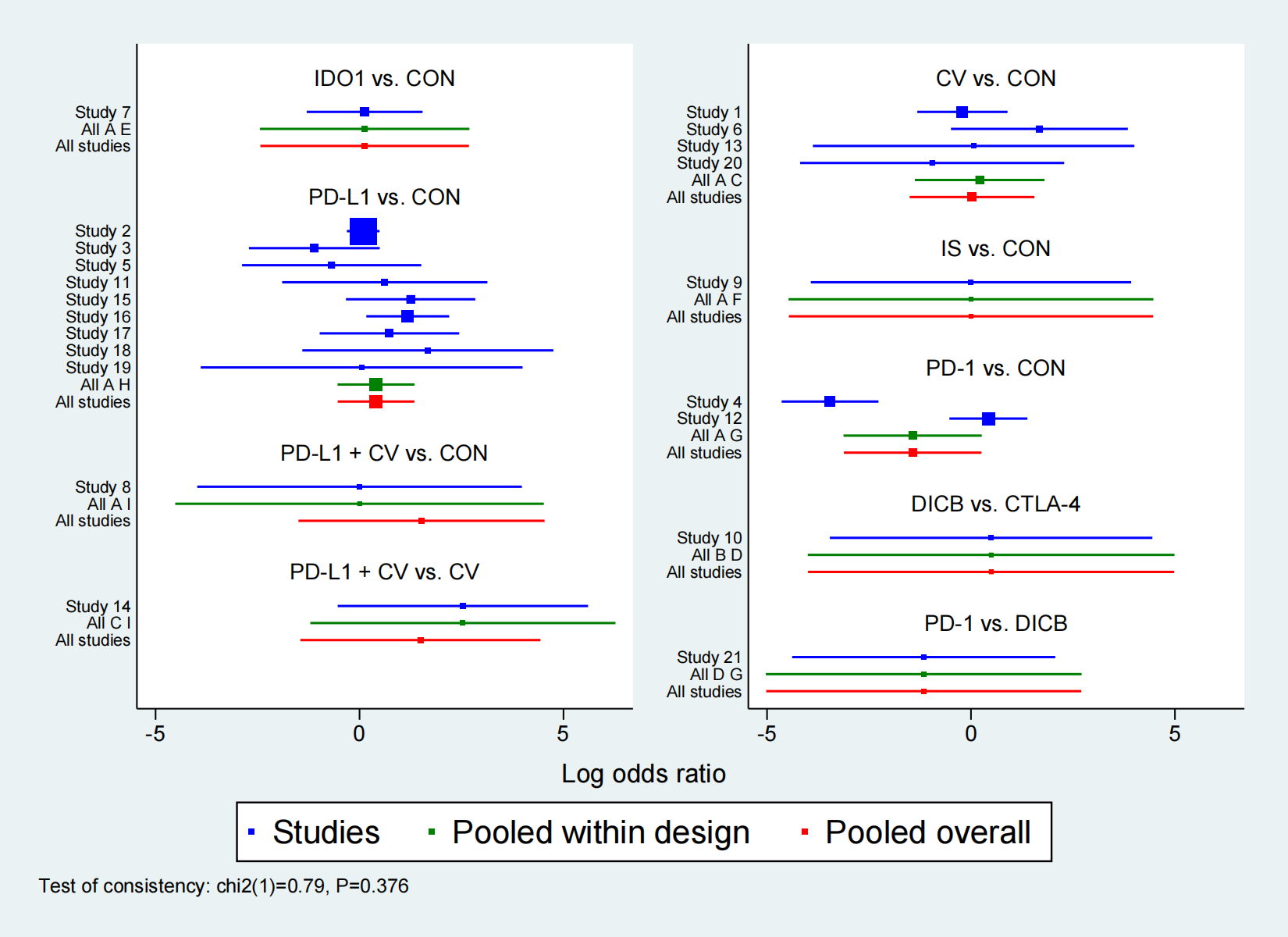


Figure 5.3. Forest plot of treatment-related adverse events (consistency test: p = 0.376).


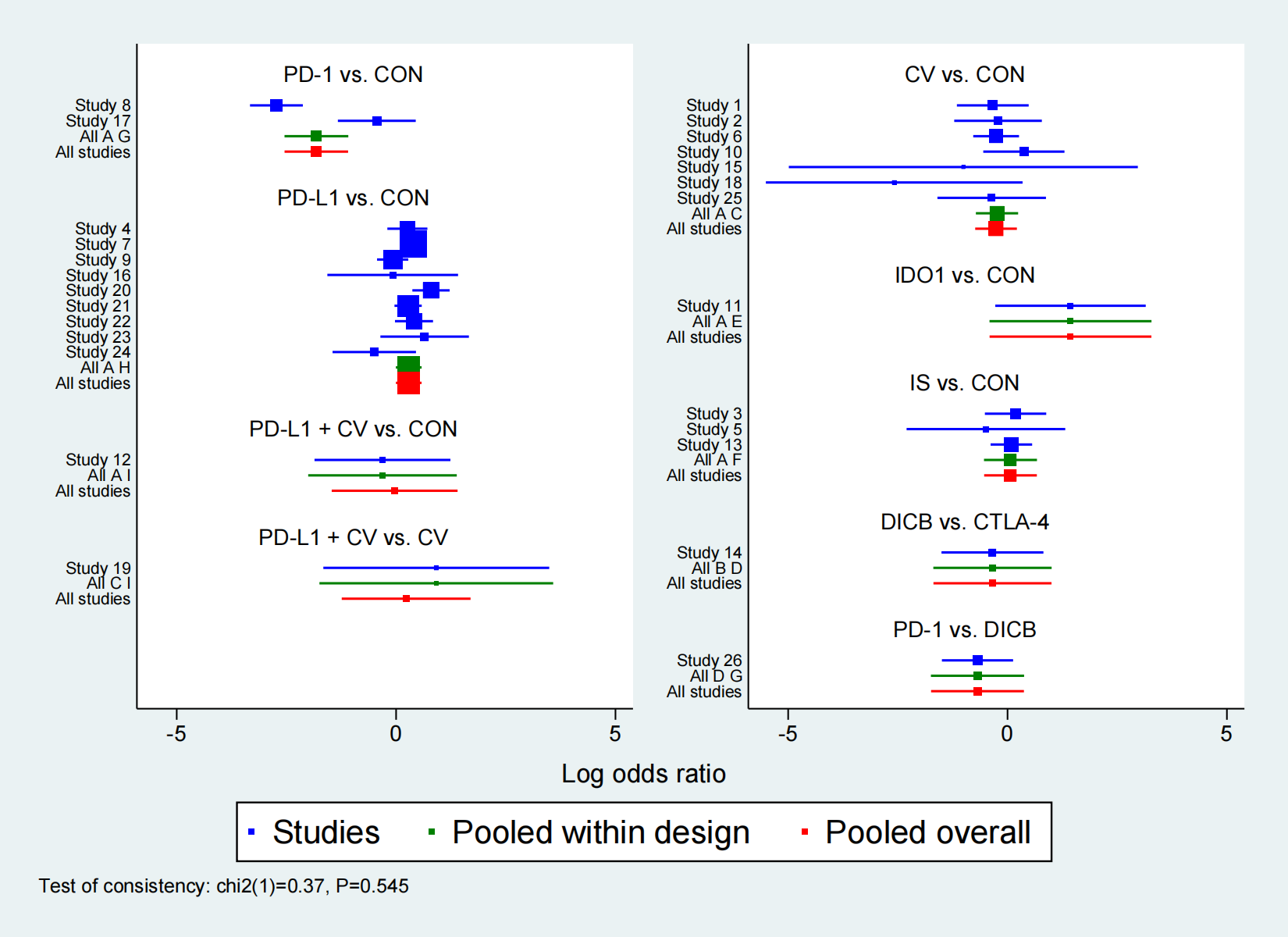


Figure 5.4. Forest plot of grade ≥ 3 treatment-related adverse events (Consistency’s test: p = 0.545).
